# Impact of similarity threshold on the topology of molecular similarity networks and clustering outcomes

**DOI:** 10.1186/s13321-016-0127-5

**Published:** 2016-03-30

**Authors:** Gergely Zahoránszky-Kőhalmi, Cristian G. Bologa, Tudor I. Oprea

**Affiliations:** Translational Informatics Division, University of New Mexico School of Medicine, MSC09 5025, Albuquerque, NM 87131 USA

## Abstract

**Background:**

Complex network theory based methods and the emergence of “Big Data” have reshaped the terrain of investigating structure-activity relationships of molecules. This change gave rise to new methods which need to face an important challenge, namely: how to restructure a large molecular dataset into a network that best serves the purpose of the subsequent analyses. With special focus on network clustering, our study addresses this open question by proposing a data transformation method and a clustering framework.

**Results:**

Using the WOMBAT and PubChem MLSMR datasets we investigated the relation between varying the similarity threshold applied on the similarity matrix and the average clustering coefficient of the emerging similarity-based networks. These similarity networks were then clustered with the InfoMap algorithm. We devised a systematic method to generate so-called “pseudo-reference” clustering datasets which compensate for the lack of large-scale reference datasets. With help from the clustering framework we were able to observe the effects of varying the similarity threshold and its consequence on the average clustering coefficient and the clustering performance.

**Conclusions:**

We observed that the average clustering coefficient versus similarity threshold function can be characterized by the presence of a peak that covers a range of similarity threshold values. This peak is preceded by a steep decline in the number of edges of the similarity network. The maximum of this peak is well aligned with the best clustering outcome. Thus, if no reference set is available, choosing the similarity threshold associated with this peak would be a near-ideal setting for the subsequent network cluster analysis. The proposed method can be used as a general approach to determine the appropriate similarity threshold to generate the similarity network of large-scale molecular datasets.

**Electronic supplementary material:**

The online version of this article (doi:10.1186/s13321-016-0127-5) contains supplementary material, which is available to authorized users.

## Background

Complex network theory based clustering algorithms represent a relatively new class of methods applied to the field of cheminformatics. This class of methods can process large data sets in reasonable time. The core of the decision making mechanism of these network, or graph theory based methods, is the connectivity matrix of the network, i.e. which nodes are inter-connected. This connection structure can be perceived as information spread across the network. This information is used for inferring what node is likely to be similar to other nodes, based on what nodes they have in common. Network clustering algorithms, which are also referred to as community or module detection algorithms, operate on a similar basis. They seek groups of similar subjects based on the node neighborhood. Examples of such algorithms are the *k*-clique percolation method (CPM) [1–3] combined with a level selection algorithm (LInCS) [4], the InfoMap algorithm [5], and the Girvan–Newman algorithm [6]. The outcome of any network based clustering is substantially influenced by the underlying network topology.

Cheminformatics networks are typically generated using the similarity matrix derived from the molecules of interest. Such networks are often referred to as *similarity networks*. The process of converting the similarity matrix into a similarity network is not obvious. Typically a threshold is applied to the values of the similarity matrix, leading to a so-called *threshold matrix* [4, 7]. Pairs of molecules are preserved as pairs of nodes connected by an edge if their similarity-coefficient is greater than or equal to the selected cut-off similarity value, denoted as *t*. This process results in an unweighted and undirected network. Ideally this network is able to highlight structural relations between the chemical structures at hand. The question arises: How can one select a threshold value so that the similarity network serves as an optimal or near-optimal input for the subsequent clustering step?

The importance of this question is apparent in the context of “Big Data”. To our knowledge, no systematic method addresses the aforementioned question. We have summarized our requirements for a systematic similarity threshold selection mechanism, which are: (1) ability to process large molecular datasets; (2) similarity measure independence; (3) use of structural chemical information only; (4) support the decision making process with well-defined network topology parameters. In the following we provide a short summary of earlier attempts that addressed the challenges, at least in part.

A common approach to converting a similarity matrix into a similarity network is to apply a threshold or series of thresholds on the similarity matrix. Saito et al. [8] used statistical significance testing to identify positively correlated pairs of molecules, which are connected by an edge in the resultant network. The emerging network topology is a function of the selected significance level. A more common approach is to apply a series of thresholds on the full similarity matrix. This approach was utilized decades ago for clustering documents based on keywords [7]. Tanaka et al. [9] used this approach in cheminformatics, however that investigation focused on small-world properties [10] of the emerging similarity networks. Wawer et al. [11] applied a series of thresholds to generate similarity networks. Their selection of the applied threshold *t* = 0.65 was driven by evaluating clusters based on available bioactivity data. As a secondary data source, bioactivity is not always available, thus this method cannot be used when only chemical structures are available; furthermore, by changing the endpoint from one bioactivity source to another, clusters are likely to re-arrange. The threshold *t* = 0.65 value is based on the drug-like MACCS fingerprints [12], which are unlikely to be suitable for analyzing datasets of Big Data given the low discrimination capacity provided by such a relatively small number of structural keys. Furthermore, Wawer et al. [11] only discuss network topology from a high-level point of view, i.e. through the number, size, density and composition of components.

Given our interest in molecular similarity networks from a clustering point of view, our attention was drawn to a promising and well-defined network topology descriptor. This descriptor is the so-called average clustering coefficient (*ACC*) [10]. The use of *ACC* in conjunction with (similarity) thresholds can be found in prior art, e.g. Serrano et al. [13] used it in the realm of physics. This study did not analyze molecular similarity networks, but some of its findings demonstrated that the *ACC* could indicate changes in network topology. Barupal et al. [14] show that the selection of the similarity threshold in metabolite networks can change the individual clustering coefficient values of nodes. Nevertheless, none of the latter two studies provide a systematic method for selecting a suitable similarity threshold. To our knowledge, it was our previous work, by Zahoránszky et al. [4], that provided a first systematic method for selecting a similarity threshold to promote the success of a subsequent network clustering step. While this method was able to inspire research [15] outside the realm of cheminformatics it was not evaluated on large molecular datasets. Otherwise, the method meets the rest of our criteria raised against a systematic similarity threshold selection method. Therefore, we extended and generalized this approach.

The scope of this study is to find a methodology-driven transformation of a similarity matrix into a network that facilitates a near optimal outcome of a particular clustering workflow. Naturally the optimal outcome will be constrained by the choice of similarity measures and clustering algorithms. The transformation should be able to handle large datasets and to operate on the basis of objective network topology measures, in order to reduce the need for making subjective decisions by the investigator.

## Datasets and methods

### Molecular libraries

Graph theory provides the underpinning of quantifying similarity between molecular structures. The atoms of molecules constitute the nodes of the graph whereas the bonds constitute the edges. The nodes and the edges are labeled according to the available chemical information, i.e. types of atoms and bonds. This representation might be referred to as a *molecule graph*. In this study molecular structures are encoded as isomeric SMILES [16] which is a widely used language to describe molecule graphs. The following subsections introduce the data sets analyzed in the present study.

#### Small combinatorial libraries

A small set of 157 molecules has been proposed [4] to be used as a reference data set for clustering studies. Molecules were manually selected utilizing expert knowledge so they can be assigned to six clusters representing the original six combinatorial libraries the compounds were synthesized in. The number of molecules selected to each reference cluster shows variation that reflects the intention of designing the reference data set. In each cluster molecules are more similar to each other than to molecules of another cluster. The reason of it is that combinatorial synthesis produces molecules that share the same core, referred to as *scaffold*. Considering that the six different combinatorial libraries represent six different scaffolds it is assured that intra-cluster similarity is greater than inter-cluster similarity. This data set will be referred to as Small Combinatorial Libraries (SCL) through this study. The original molecular structures of SCL were deposited by AMRI Inc. (former Comgenex) [17] in the ZINC 7 database [18].

#### WOMBAT 2010 data set

The World of Molecular Bioactivity (WOMBAT) database (version 2010) [19] is a manually curated comprehensive biological activity database for small molecules. It comprises 300,000 unique molecular structures, 19,000 unique targets and more than 1,000,000 biological activity data that were experimentally determined between small molecule-target pairs. Each biological activity entry is referenced by the original paper in which the experimental result was reported. Small molecules were extracted from the WOMBAT database in the form of isomeric SMILES. Next, a standardization scheme was applied on the structures which is described in details in subsection “Structure standardization”. Removal of any duplicate structures resulted in 244,143 unique molecular structures.

#### PubChem MLSMR data set

The PubChem Molecular Libraries Small Molecule Repository (MLSMR) [20–24] is a library that was designed for facilitating high-throughput screening campaigns. The library contains several distinguished subsets such as (1) known bioactive compounds such as toxins and drugs, (2) natural products, (3) compounds focused on a variety of biological target families and (4) large number of compounds attributing to a significant diversity. The size of the library has evolved in multiple cycles to achieve a number of 400,000 compounds the time the experiments of this study were carried out. Therefore, this data set provides a large and diverse sample of known and potential bioactive chemical space. Furthermore, the data set contains large number of smaller subsets that can be considered as structure-activity relationship (SAR) series. This unique balance of diversity and structural relatedness make this library useful for lead identification and optimization. After standardization and duplicate filtering: 353,028 unique structures.

### Structure standardization

The SCL dataset has been imported into ChemAxon InstantJChem (version 5.7.0) [25]. Next, the molecules were extracted from the database as canonical SMILES using the “smiles:au-H” formatting string. The exported structures were object to another standardization in the pipeline which contains a “keep largest fragment only” and a “general” aromatization steps. These standardization steps were performed using the ChemAxon’s *standardize* utility from the JChem library (version. 3.2.10).

The WOMBAT and PubChem MLSMR datasets were imported into a ChemAxon InstantJChem database (version 5.3.8). The structures were exported from this database as canonical SMILES using the “smiles:au0-H” formatting string. The extracted structures were subject to another standardization step with the help “*standardize*” utility of ChemAxon’s JChem library (version: 5.3.6) using the -*c “keepone*..*neutralize*..*aromatize*..*[O*−*][N*+*]*=*O*>>*O*=*N*=*O*..*N*=*[N:1]#[N:2]*>>*N*=*[N*+*:1]*=*[N*−*:2]”* −*f “smiles:au0*-*Hn”* parameters. The duplicate structures were removed.

### Similarity measures

A family of techniques utilized to quantify similarity between molecules starts with extracting structural features as subgraphs from the graph of molecular structures. The set of extracted structural features will characterize a molecule. This set of features is often referred to as a *topological fingerprint* [26, 27]. The more features two molecules have in common the more similar they are [28, 29]. In this study three major types of molecular fingerprints were used, namely structural key fingerprints, hashed binary fingerprints and extended connectivity fingerprints (ECFP). Structural key based fingerprints were computed using the Open Babel (version 2.3.2) implementation [30] of the original MACCS keys [12]. It should be noted that only 122 out of the original 166 MACCS keys is used in the Open Babel implementation due to the unavailability of the rest of the original MACCS keys. Hashed binary fingerprints of length 1024, 2048 and 4096 were generated by using ChemAxon’s GenerateMD utility (version 3.2.10) [25, 31]. Extended connectivity fingerprints of diameter 4, 8, and 12 were generated by an in-house implementation of the underlying algorithm [32–34]. In correspondence with the predefined diameter *d*, types of ECFPs are distinguished by suffixing the abbreviation with the applied parameter *d*; ECFP_4 refers to a fingerprint in which the diameter of the extended neighborhoods is 4. Although in the main body of this study molecules were characterized by ECFP_4 fingerprints, some of the results were obtained by using ECFP_8 and ECFP_12 fingerprints.

With the help of molecular fingerprints it is possible to quantify the similarity between molecules. This step requires the application of a so-called *similarity measure*. The *Tanimoto similarity*-*coefficient* [35] is one of the most widely used similarity measures in cheminformatics. The idea of this metric is to express the ratio of the common and distinct structural features of two molecules. Accordingly, the maximal value of the Tanimoto similarity-coefficient is 1 whereas the minimal is 0 corresponding to highest and lowest similarity, respectively. As described above, several methods exist to capture structural characteristics of molecules in a form of molecular fingerprints. In the case of fingerprints of fixed length, e.g. MACCS-fingerprint and ChemAxon hashed binary fingerprints, computing the Tanimoto similarity-coefficient *T*(*m*_*A*_,*m*_*B*_) is performed according to *Formula*1, where *A* and *B* denote the set of indices of bits with a value of 1 in the fingerprints of molecule *m*_*A*_ and *m*_*B*_, respectively.

1 $$T\left( {m_{A} ,m_{B} } \right) = \frac{\left| A \right| \cap \left| B \right|}{\left| A \right| \cup \left| B \right|}$$

The means of computing Tanimoto similarity-coefficient between extended connectivity fingerprints follows a similar logic. Considering that ECFPs are comprised of integers instead of bits, moreover the length of ECFPs might vary due to the fingerprint generating algorithm, it is necessary to convert these fingerprints into a fixed-length bit-vector. One of the means to do so is treating the integers as indices of a virtual fingerprint of length *W* that corresponds to the largest integer appearing in any of the fingerprints. In agreement with this interpretation each integer represents a bit turned to 1 in a *W*-bit length virtual fingerprint. With the aid of this transformation the Tanimoto similarity-coefficient of two ECFPs can be computed as described above.

We used ChemAxon’s JChem 5.7.1 library to compute Tanimoto similarity-coefficients in the case of MACCS keys and ChemAxon hashed binary fingerprints. In the case of ECFPs an in-house developed software was used to compute Tanimoto similarity-coefficients.

### Molecular similarity network generation

Pairwise similarities between a set of molecular structures *M* defines a *similarity matrix****S*** that is a *|M|* × *|M|* squared matrix. Furthermore, ***S*** is symmetric considering that in this study the similarity of molecules is expressed as Tanimoto similarity-coefficient (see: *Tanimoto similarity*-*coefficient* above). An element *s*_*i*,*j*_ ∈ $\mathbb{Q}$ [0,1] of ***S*** represents the Tanimoto similarity-coefficient *T*(*m*_*i*_, *m*_*j*_|∀*m* ∈ *M*) defined between molecules *m*_*i*_ and *m*_*j*_. This similarity matrix can be transformed into a fully connected network constituted by molecules as nodes and edges connecting them. An edge in this network is weighted and represents the similarity relation *s*_*i*,*j*_ between the two endnodes, i.e. molecules *m*_*i*_ and *m*_*j*_ provided that *i* ≠ *j*. The weight of an edge equals to *s*_*i*,*j*_. Considering that Tanimoto similarity-coefficient is a symmetric similarity measure the edge between two nodes is undirected. Therefore this network is a weighted and undirected network. However, the topology of a fully connected network provides little help in finding interesting relations between molecules based on network topology.

A possible solution for highlighting important similarity relations is to apply a similarity threshold *t* on the original similarity matrix ***S***. Applying *t* on ***S*** will transform a similarity-coefficient to 1 if its value is greater than or equal to *t*. Otherwise the similarity-coefficient will be transformed to 0. The resultant matrix of the thresholding step is referred to as a *threshold matrix* [7] and is denoted by ***Z***. Please note that the dimensions of ***Z*** are the same as that of ***S***. Elements of ***Z*** are denoted by *z*_*i*,*j*_ ∈ {0,1} and are computed according to *Formula*2.

2 $$z_{i,j} = \left\{ {\begin{array}{*{20}l} {0, \quad s_{i,j} < t} \\ {1, \quad s_{i,j} \ge t} \\ \end{array} } \right.$$

**Fig. 1:**
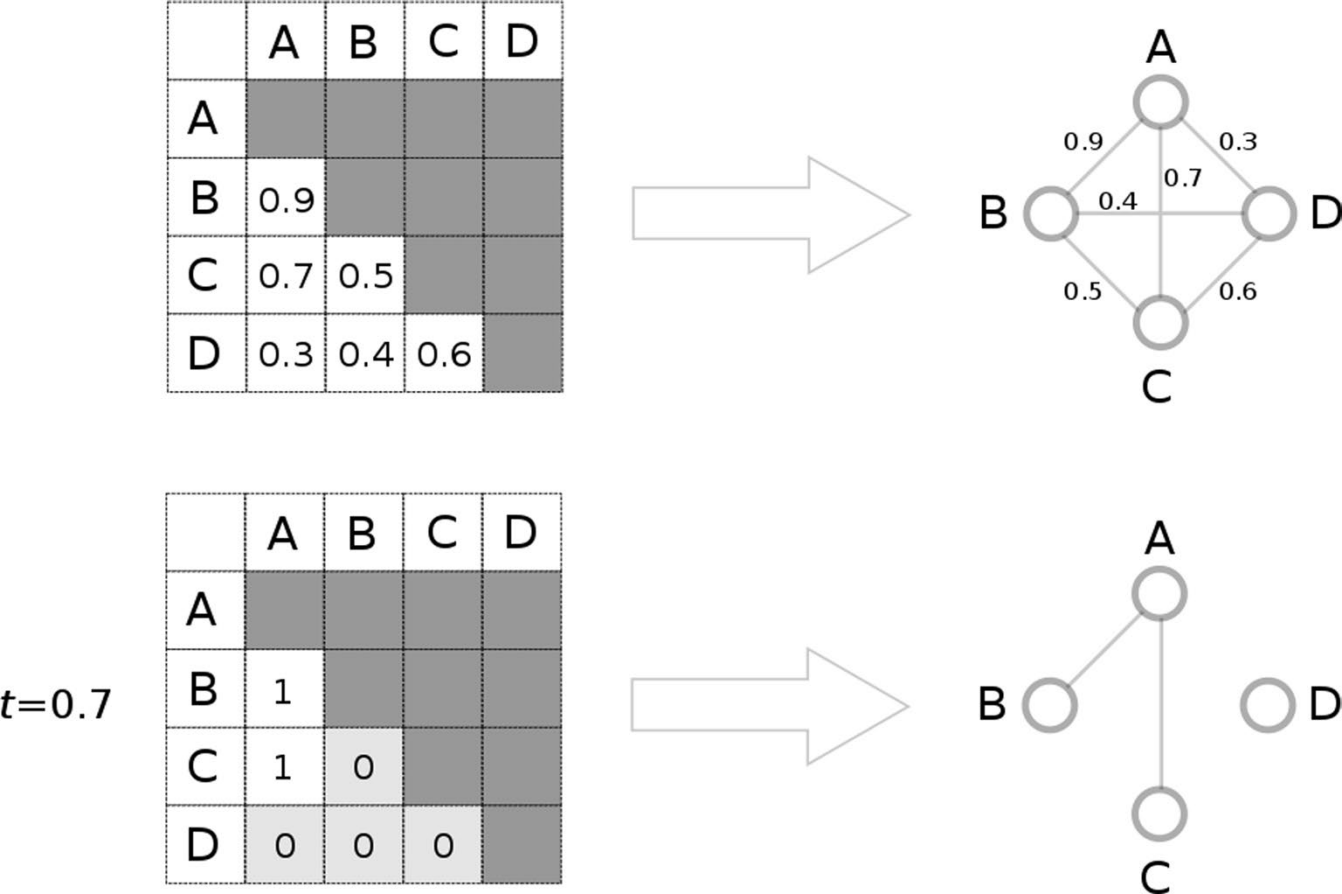
Transforming a similarity matrix to a similarity network. The upper part of the figure shows the original similarity matrix and a network representing it. The *lower part* of the figure shows a threshold matrix and the corresponding similarity network that was derived by applying a t = 0.7 similarity threshold on the original similarity matrix. Elements of the similarity matrix containing similarity-coefficients greater than or equal to t = 0.7 are transformed to 1. Rest of the elements of the similarity matrix are colored with light gray in the threshold matrix and their values are transformed to 0. In the resultant similarity network molecule D is a singleton because it only has molecules less similar to itself than the similarity threshold of choice

Threshold matrix ***Z*** can be transformed into a network by similar means as the similarity matrix. However, according to our initial aim, i.e. to highlight important similarity relations based on the topology merely, there is no need to preserve the weight of the edges. This transforms the initial meaning of an edge into a new binary relation: the existence of an edge between two nodes represent a *T*(*m*_*i*_, *m*_*j*_) ≥ *t* similarity relation between molecules *m*_*i*_ and *m*_*j*_. The network can be readily derived from ***Z*** as follows. If *z*_*i*,*j*_ = 1 then an edge is defined between nodes representing molecule *m*_*i*_ and *m*_*j*_. On the other hand, if *z*_*i*,*j*_ = 0 then no edge is defined between the corresponding nodes. The resultant network is therefore unweighted and undirected and can be referred to as a *similarity network*. It should be noted that similarity matrix **S** might contain molecules that only have Tanimoto similarity-coefficients lower than the applied threshold. This kind of molecules will only have zeros in the corresponding row in the threshold matrix ***Z***. In similarity networks such a molecule is represented as a single node, i.e. a *singleton*. The process of generating similarity networks is illustrated in Fig. 1.

### Average clustering coefficient

Let *G* = (*V*, *E*) denote a network constituted by a set of nodes *V* and a set of undirected and unweighted edges *E* (*U* × *V*)|*u*, *v* ∈ *V*, ∀(*u*, *v*) : *u* ≠ *v* connecting the nodes. A node *v* ∈ *V* is considered a neighbor of node *i* ∈ *V* if (*i*, *v*) ∈ *E*, i.e. an edge exists between the two nodes. The degree *deg*(*i*) ∈ $\mathbb{N}$ of node *i* is defined as the number of edges associated to node *i*.

Let *N*(*A* × *B*) ⊆ *E*|*A*, *B* ⊆ *V*\*i*, ∀*a* ∈ *A* : (*i*, *a*) ∈ *E*, ∀*b* ∈ *B* : (*i*, *b*) ∈ *E* denote a subset of edges that connect the neighbors of node *i*. Please note, that none of the edges between node *i* and its neighbors is member of this edge subset *N*.

The *clustering coefficient*, denoted by *CC*(*i*) ∈ $\mathbb{Q}$ [0,1] of a node *i* ∈ *V* in the network *G* is defined as the ratio of the number of existing edges between the neighbors of node *i* and the number of possible edges between its neighbors [10]. If node *i* has none or only one neighbor then *CC*(*i*) = 0 by definition.

Using the above introduced concepts the formal definition of clustering coefficient is given by *Formula* 3.

3 $$CC\left( i \right) = \left\{ { \begin{array}{*{20}l} {0,\quad \deg \left( i \right) \in \left\{ {0,1} \right\}} \\ {\frac{2\left| N \right|}{{\deg \left( i \right)\left( {\deg \left( i \right) - 1} \right)}},\quad \deg \left( i \right) > 1} \\ \end{array} } \right.$$

It can be seen that the clustering coefficient is a local parameter that provides information on the local topology of a particular node. On the other hand, the *average clustering coefficient ACC*(*G*) ∈ $\mathbb{R}$[0,1] is a global parameter that characterizes the overall network topology of *G* [10]. It takes into account the clustering coefficient values of the individual nodes that have a degree greater than zero. Let *X* ⊆ *V*|∀*x* ∈ *X* : *deg*(*x*) > 0 denote the subset of such nodes. Accordingly, the average clustering coefficient is defined formally by *Formula* 4.

4 $$ACC\left( G \right) = \left\{ { \begin{array}{*{20}l} { 0,\quad \left| X \right| = 0} \\ {\frac{1}{\left| X \right|}\mathop \sum \limits_{i = 1}^{\left| X \right|} CC\left( {x_{i} } \right),\quad \left| X \right| > 0} \\ \end{array} } \right.$$

### The interplay between the average clustering coefficient and the addition or removal of edges

**Fig. 2:**
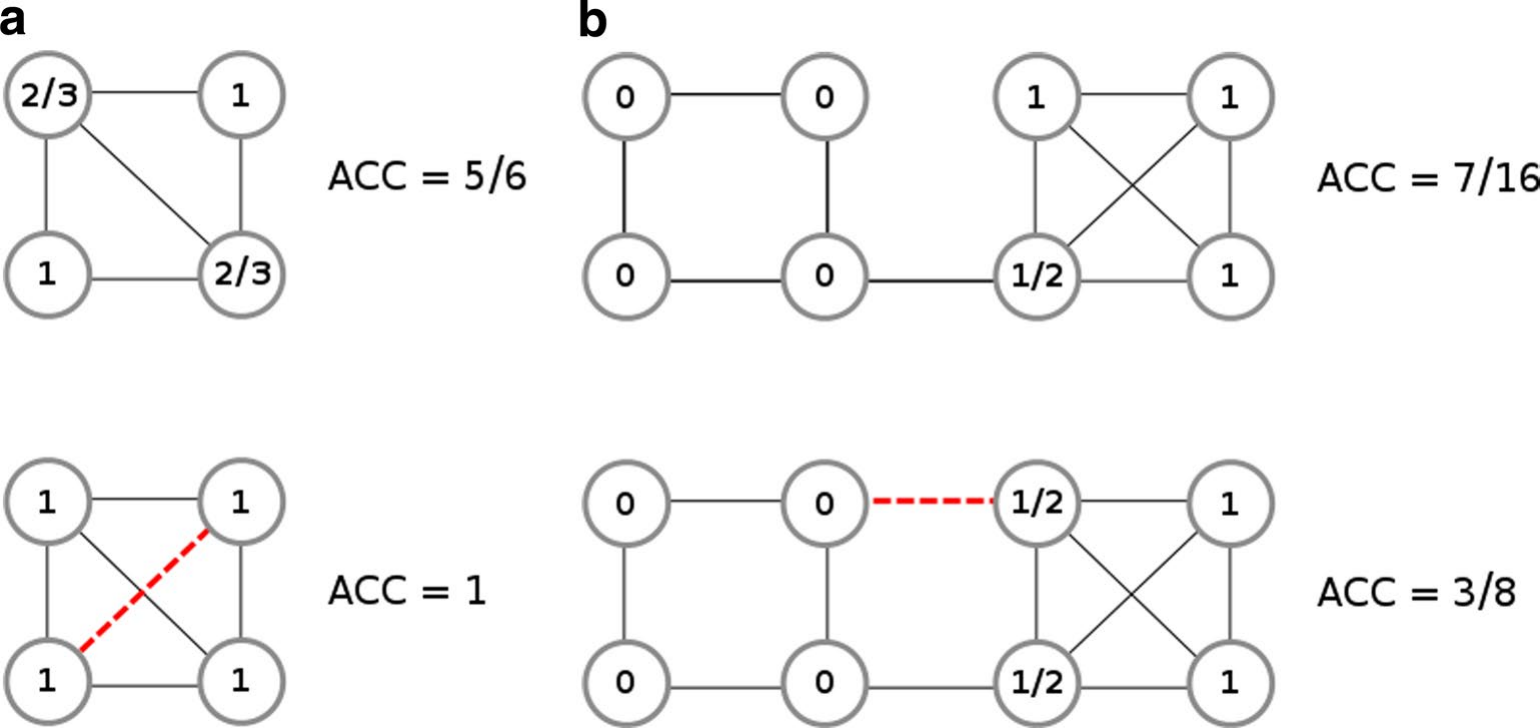
The influence of edge addition/removal on the average clustering coefficient. An intriguing dynamics between a network’s average clustering coefficient is observed upon adding or removing edges from the network. **a** Provides an example in which the average clustering coefficient increases followed by the addition of a new edge, shown as *red dashed line* in the lower network. **b** Shows a somewhat counterintuitive scenario in which the average clustering coefficient of a network actually decreases upon the addition of one edge. The added edge is shown as *red dashed line* in the lower network

The ACC of a network is subject to change in case of edge addition or edge removal. The dynamics of this process is quite intriguing: one would expect that addition of new edges to an existing network would increase the connectedness. While this is true, i.e. more nodes will become connected, it does not follow that the existing neighbors of a node are more likely be connected. Acquiring a new neighbor upon an edge addition does not increase the clustering coefficient of the host node if the new neighbor won’t be connected to any of the already existing neighbors of the host node. Furthermore, removal of an edge can actually lead to an *ACC* increase. The changes described here are illustrated with examples in Fig. 2.

The phenomenon described above can be observed when a series of similarity networks are generated from a given similarity matrix by applying a series of thresholds. Increasing the threshold implies removing edges from the network. Applying a strictly monotonically increasing series of thresholds on a similarity matrix does not necessarily lead to a strictly monotonic decrease in the edge number of the generated similarity networks. This happens if an increment in the threshold does not meet the value of the next lowest Tanimoto similarity-coefficient of a pair of molecules. In this case the two networks generated by the previous and the incremented thresholds will be identical despite the threshold value increase.

### Clustering framework and performance analysis

Evaluating clustering performance is still a challenge to date for a number of reasons. First of all the number of available reference sets, often referred to as *ground truth* sets, is very limited. A common reason is that the data set at hand is proprietary in nature. Even though the data set might be accessible, the lack of exact definition of a cluster per se contributes some extent of inherent subjectivity to the process of determining which object belongs to which cluster, i.e. to the creation of *reference clustering*.

One of the common strategies to define a reference clustering for a set of molecular structures requires the involvement of *expert knowledge*. In this process a chemist would inspect individual molecular structures and assign them to clusters based on a predefined clustering objective. This human-dependent approach becomes cumbersome, then intractable as the data sets reach the thousands range. To overcome this barrier a *computer aided method* is required to substitute expert knowledge in the process. A plausible way to achieve this is to apply an adequate combination of a pattern-recognition algorithm and a clustering algorithm. Considering that numerous pattern recognition and clustering algorithms exist it is likely that the resulting clustering will be different in each case, although some degree of consensus might be expected.

In the following subsection we describe a clustering framework that was used to analyze the effect of choosing a certain similarity threshold on the clustering performance. The clustering framework consists of (a) three reference clustering sets, (b) a clustering algorithm and (c) a performance evaluation method. It should be noted, however, that the aim of cluster analysis was *not* to achieve the ideal clustering in light of the reference sets, but to show how the choice of similarity threshold influences the performance when the same clustering workflow and reference clustering sets are used for comparison. For this reason we accept that one might argue for the existence of other means to create the reference clustering sets and to perform the cluster analysis. Nevertheless, the clustering framework assures that the observed variance in the clustering performance is accounted *solely* for the choice of similarity threshold. This holds true, because the applied reference clustering sets and the clustering algorithm are consistent through the entire study.

#### Reference clustering data sets

As mentioned above there exist various approaches to generate a reference clustering set. A specimen of a reference clustering set generated by an expert is the SCL dataset [4]. In this set 157 molecules are assigned to six different clusters that correspond to six clearly defined scaffolds shared by the members of each cluster. For further information on the SCL data set please refer to the subsection ‘Molecular libraries’. Results obtained by using the SCL data served the purpose of proof-of-concept. However, we felt it necessary to investigate data sets that better reflect the size of common chemical libraries. To this end, in this study we analyzed additionally the WOMBAT [19] and the PubChem MLSMR datasets [20]. Considering that no known reference clustering exists for these data sets we needed to overcome several challenges to generate those.

The number of molecular structures contained by the WOMBAT and PubChem MLSMR data sets is in the range of hundreds of thousands. Therefore the possibility of clustering the molecules relying on expert knowledge was ruled out. Instead, we have devised a computer aided method to generate a so-called *pseudo*-*referenceclustering* for the datasets. The method of generating reference clusters is described in details as follows.

We devised a two-phase procedure to generate the pseudo-reference clustering for the two large datasets. In the first phase, an in-house implemented algorithm operates on the basis of a well-defined clustering objective. This objective follows a chemical rule-set that was designed to mimic the decision-making process of a medicinal chemist in identifying common structural features of molecules. To this end, the algorithm searches for so-called *maximal common edge subgraphs* (*MCESs*) [36] with the help of a modified version of the RASCAL-algorithm [37]. In the implementation of the algorithm an MCES is allowed to be constituted by multiple disconnected subgraphs. The algorithm utilizes two major heuristics based solutions to make the clustering capable to handle large datasets. One of these solutions is to decompose the molecules according to the hierarchical scaffold (HierS) decomposition algorithm [38, 39]. The HierS sets enable to eliminate the analysis for pairs of molecules if the differences between these sets indicate the lack of common ring systems. If the HierS sets don’t exclude a pair of molecules from MCES analysis then a second heuristic is applied to potentially identify an MCES. To this end, molecules that contain less than 40 heavy atoms [16] are analyzed by an exact MCES finding algorithm. Molecules having between 40 and 80 heavy atoms are passed to an algorithm that utilizes a certain approximation in identifying MCES. Molecules with more than 80 heavy atoms were excluded from the MCES analyses due to performance limits. Once MCESs are identified, each MCES will represent one cluster and the cluster will be comprised of molecules that contain the particular MCES. The members of the clusters will only differ in the so-called *linkers* and *R*-*groups* that separate and/or augment the parts of MCES, respectively. This sort of decomposition of molecular structures, i.e. MCES, linkers and R-groups, follows a common practice in the field of medicinal and computational chemistry. The resulting MCES-clusters are typically small in size and the members are in a rigorous, medicinal chemistry based structural relation with each other.

One characteristic of the generated MCES-clusters is that the structures of cluster members might contain twice as much, or even more heavy atoms as the MCES of the cluster. In line with our original aim, i.e. to generate well defined clusters, we thought it necessary to apply an extra filtering step on the MCES-clusters. Therefore, in the second phase of the process certain clusters including all the cluster-members were eliminated from the dataset. The criterion for eliminating a cluster is based on the heavy-atom count of the MCES and cluster members. If *any* cluster member harbors a heavy-atom count that exceeds that of the MCES by more than two-fold, then the entire cluster is eliminated.

The filtering step of the second phase is necessary in order to maintain a certain level of structural coherence within an MCES cluster. Otherwise, it may happen that two molecules share the same MCES consisting of two disconnected heterocycles that are connected through a much larger ring system, which might be different in the two molecules. In this case the validity of assigning the two molecules to the same MCES cluster might be questioned. This filtering step does not incorporate a definite similarity constraint on the members of an MCES cluster. The Tanimoto similarity-coefficient between members might very well be under 0.5, a value that intuitively might occur in connection with the applied filtering step described above.

**Fig. 3:**
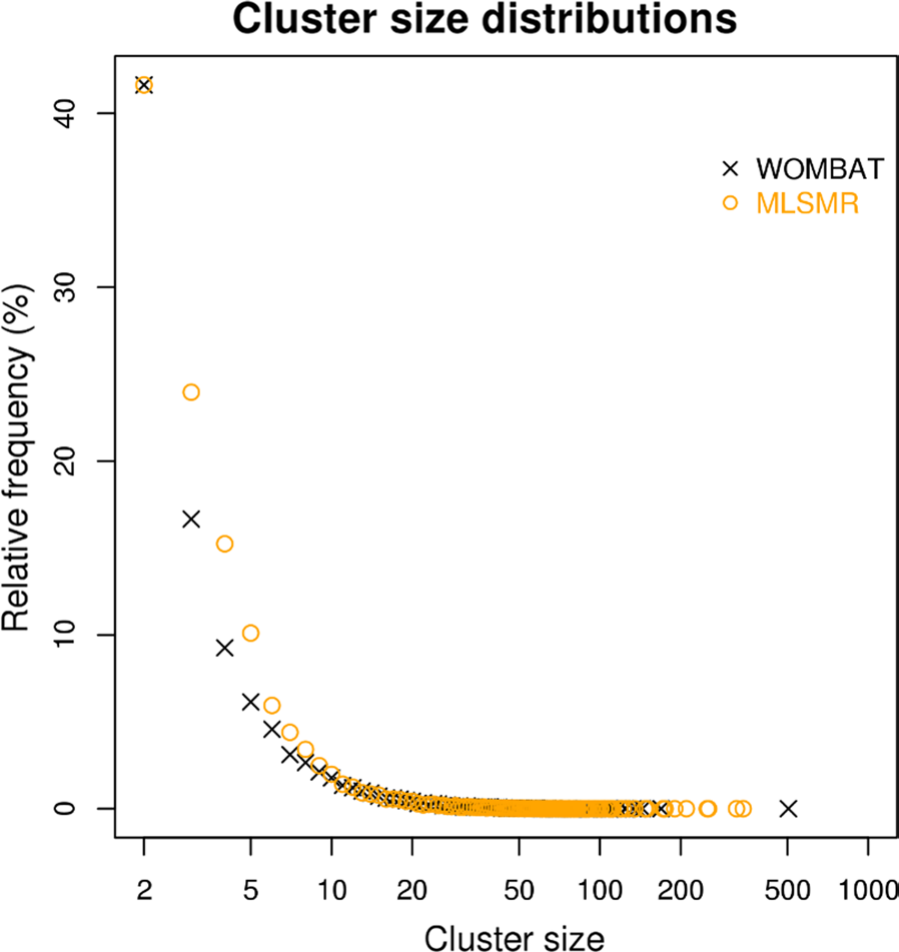
Cluster size distribution of pseudo-reference clustering datasets. The x-axis of the graph is shown on log-scale and it represents the size of clusters in the case of the pseudo-clustering datasets generated from the WOMBAT and PubChem MLSMR datasets. The y-axis represents the relative frequency of certain cluster sizes. A given dataset is characterized by cluster sizes that have a higher frequency. The overall frequency of cluster sizes provides the cluster size profile of a dataset. As it can be seen the cluster size profile of the two datasets are nearly identical, with small differences in the low cluster size and in the large cluster size regions

The above steps gave rise to the pseudo-reference clustering datasets derived from the original WOMBAT and PubChem MLSMR datasets. The WOMBAT-derived set contains 154,012 molecules in 27,168 clusters whereas the PubChem MLSMR-derived set contains 276,960 molecules in 52,287 clusters. The distribution of cluster sizes in these two pseudo-reference clustering datasets is shown on Fig. 3.

While it is true that clusters were generated through an automated process, the clustering objective was underpinned by a rigorous, medicinal chemistry based structural rule set. Therefore, we find the pseudo-reference cluster generating scheme a useful and feasible alternative for the tedious process of defining reference clustering manually by an expert.

#### The InfoMap clustering algorithm

In this study we utilized the InfoMap [5] network-based clustering method which is able to process a threshold matrix of molecules. The reasons of selecting the InfoMap algorithm as the clustering method of this study are as follows. In a thorough clustering review by Fortunato [40], the InfoMap algorithm was shown to have one of the best overall performance investigating a variety of input datasets. Also, the InfoMap algorithm scales well with the problem size which is one of its most important characteristics in the light of the objective of this study. Furthermore, it requires minimal number of input parameters from the user. Finally, the number of clusters and the members are determined by the algorithm. These traits make the outcome of the cluster analysis rather independent from a subjective bias potentially introduced by the user. Another important property of the produced clustering that clusters are non-overlapping. We carried out the InfoMap clustering experiments using the implementation published by the authors of the algorithm (version: July 26, 2010). When performing the InfoMap clustering, in the case of each dataset, we applied a value of 1000 as the parameter for the number of iteration cycles.

#### Evaluating clustering performance

In this study we decided to apply the widely utilized sensitivity and specificity measures [41] to quantify clustering performance. The minimal and maximal values of these measures are 0 and 1, respectively. In the case of an ideal clustering both measures have the value of 1. Hence, the closer the actual values of sensitivity and specificity are to 1 the closer the actual clustering approaches the ideal one. The formal definition of sensitivity and specificity is provided in *Formula* 5, where *TP*, *FP*, *TN* and *FN* stand for the number of true positives, false positives, true negatives and false negatives, respectively. Please note, that no singletons are present neither in the reference, nor in the pseudo-reference clustering datasets, therefore the sensitivity and specificity computation will always lead to a rational number in the range of 0 and 1.

5 $$sensitivity = \frac{TP}{TP + FN}, \quad specificity = \frac{TN}{TN + FP}$$

Although the computation of the above measures is quite simple, the large size of the WOMBAT and PubChem MLSMR datasets required a specific implementation in order to achieve a reasonable runtime. This implementation relies on two important software design elements that will be discussed briefly: (1) clustering is represented as *cluster membership lists* (*CMLs*) that resembles the well-known adjacency list data structure, (2) set operations are utilized on the CMLs to efficiently compute the values of *TP*, *FP*, *TN* and *FN*.

Clustering is represented by the CMLs as follows. Each list of the CMLs starts with the identifier of the node, referred to as *list root*. This node identifier is followed by the identifiers of other nodes that belong to the same cluster as the list root. Compared to a more conventional clustering representation, e.g. adjacency matrix, the speedup of processing time when using the CMLs data structure is profound. This can be accounted for the observation that clustering at reasonable similarity thresholds gives rise to sparse CMLs, i.e. list roots associate to a number of nodes that are only a fraction of the size of the whole dataset. Although it is not a unique feature of the CMLs data structure, it is worth emphasizing its capacity to facilitate the handling of overlapping clusters. This feature is not exploited in this study, since the InfoMap algorithm produces only disjoint clusters.

## Results and discussion

### ACC as function of similarity threshold

We studied ACC in three datasets, namely SCL, WOMBAT, and MLSMR. In the case of the SCL dataset, the threshold starts at *t* = 0, whereas in the case of the latter two datasets it starts from *t* = 0.30. The reason of this is that computing the complete similarity matrix, i.e. setting the threshold to *t* = 0, for the WOMBAT and MLSMR datasets was intractable at the time the experiments were performed. In all cases, the upper limit of threshold was *t* = 1, and *t* was incremented in the steps of 0.01.

**Fig. 4:**
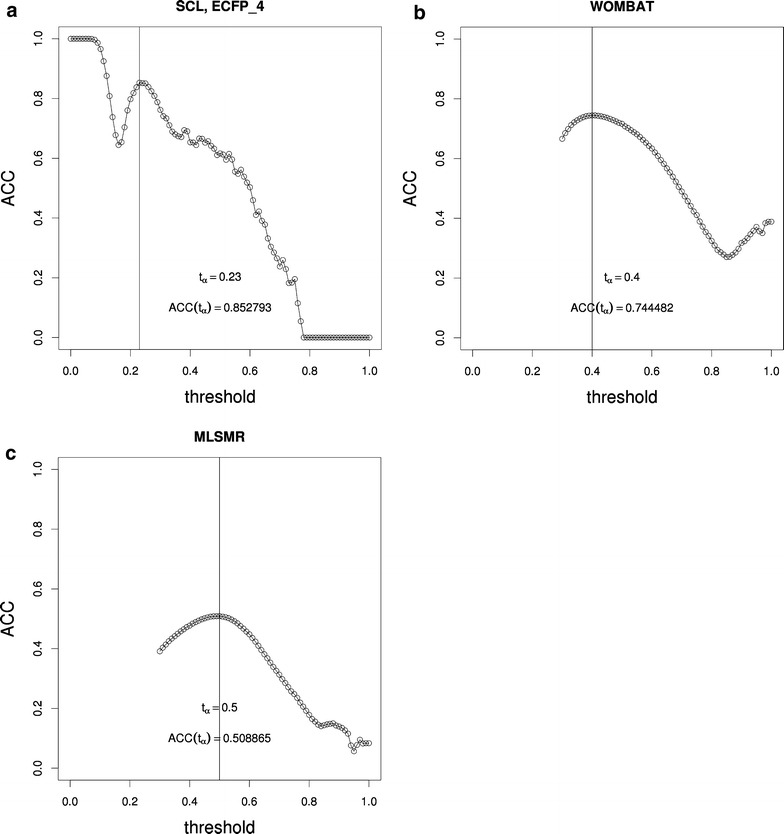
Average clustering coefficient of similarity networks in the function of the similarity threshold. For all datasets it is possible to identify a peak that stands out in comparison with the others by spanning the largest range of similarity threshold *t*. The threshold associated with the highest *ACC* value in the peak is denoted as *t*_*α*_, i.e. the so-called obvious local maximum of the *ACC*(*t*) function. Fingerprint: ECFP_4, similarity measure: Tanimoto similarity-coefficient. **a** SCL dataset. **b** WOMBAT dataset. **c** PubChem MLSMR dataset

**Fig. 5:**
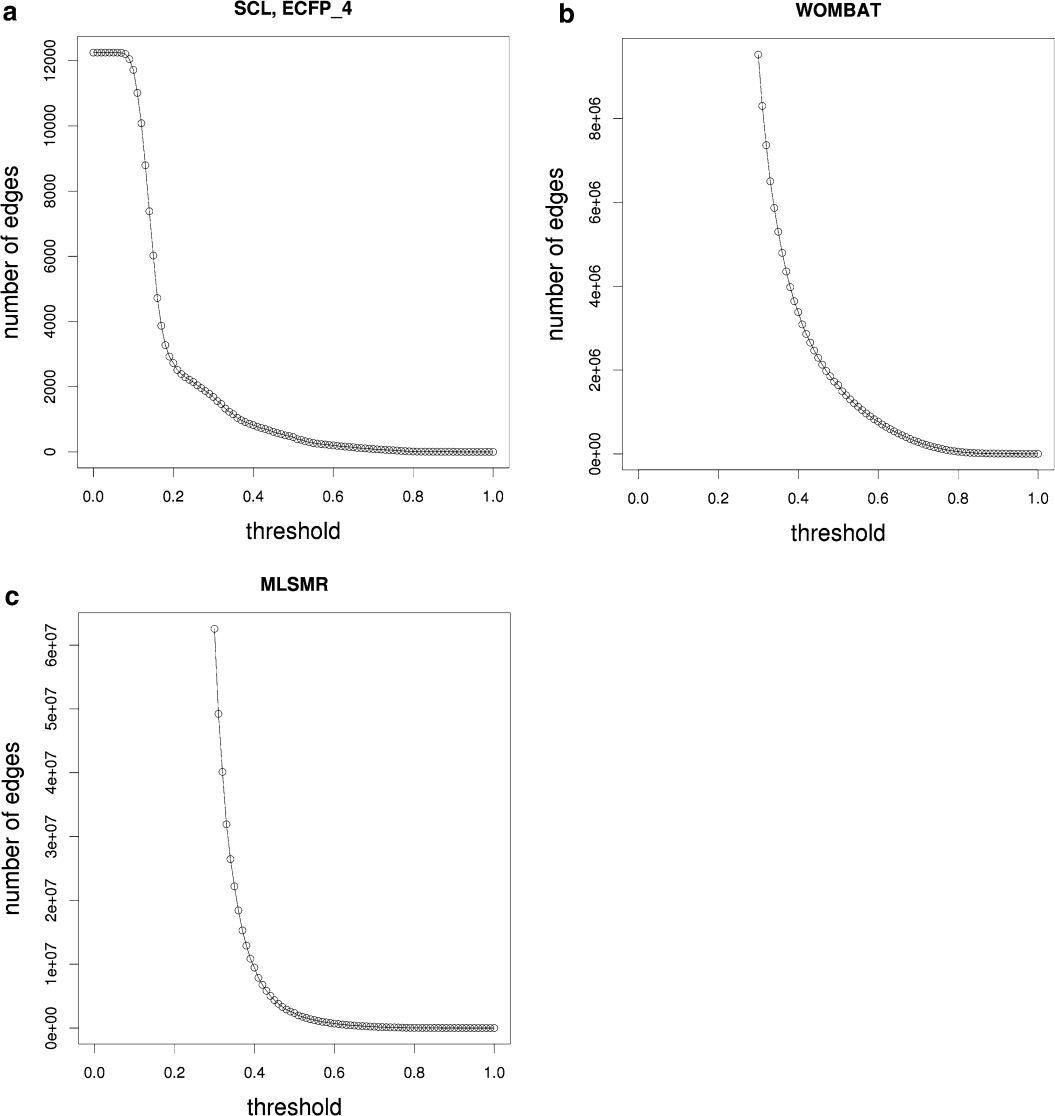
Number of edges in the function of the similarity threshold. Fingerprint: ECFP_4, similarity measure: Tanimoto similarity-coefficient. For each dataset it can be observed that the number of edges shows a decrease of steep slope at low ranges of the applied similarity threshold. This steep decline is followed by a drastic change in the slope over a short range of the similarity threshold. **a** SCL dataset. **b** WOMBAT dataset. **c** PubChem MLSMR dataset

First, we discuss our proof-of-concept SCL dataset which enabled us to make important observations. If the threshold is set to *t* = 0 the similarity network is a fully connected network, because all elements of the similarity matrix are turned to 1, hence encoding the presence of an edge between all pair of molecules (see: Fig. 4a). By definition the *ACC* of such a network is 1, as the likelihood of neighbors of a host node being connected is maximal. Therefore, setting the threshold to *t* = 0 will result in the maximal average clustering coefficient and number of edges in the respective functions *ACC*(*t*) and *EN*(*t*). It can be seen that increasing the threshold stepwise will not affect the *ACC* initially, but later it will start to decrease steeply until it reaches a local minimum. This local minimum is followed by a local maximum at *t* = 0.23. From hereafter, the threshold associated to the local maximum of the *ACC*(*t*) function is denoted by *t*_*α*_. After *t*_*α*_, the curve decreases and eventually reaches *ACC* = 0. A few shallow local maxima are observed in the range of *t* > 0.23 but their presence was not deemed important. The local maximum seems to directly follow an interesting phenomenon in the *EN*(*t*) function (see: Fig. 5a). The number of edges start to decrease steeply, then at a certain value the rate of decrease becomes slower, leading to a slight decline. The steep decrease and the sudden change in the slope of the curve is aligned with the local *ACC* maximum at *t* = 0.23. Analysis of the SCL dataset with different similarity measures provides more evidence to support this observation (see Additional file 1: Fig. S1, Additional file 2: Fig. S2, Additional file 3: Fig. S3, Additional file 4: Fig. S4, Additional file 5: Fig. S5, Additional file 6: Fig. S6, Additional file 7: Fig. S7).

To rule out that the above observations are not specific to the proof-of-concept dataset, we performed the same analysis on the larger, more complex datasets, i.e. WOMBAT and MLSMR. A local maximum of the *ACC*(*t*) function is observed for both the WOMBAT and MLSMR datasets, as shown in Fig. 4b, c, respectively. In accordance with the SCL dataset, the change in the slope of the number of edges versus threshold curve is well aligned with the local maximum of the *ACC*(*t*) function (see: Fig. 5b, c).

Furthermore, *t*_*α*_ is shifted in comparison with the SCL dataset, and differs with each dataset. The peak enclosing *t*_*α*_ in the case of WOMBAT and MLSMR follows a more elongated, flat curvature compared to the SCL dataset.

These curve characteristics unveil important differences on the underlying relations between the network objects. The local *ACC*(*t*) maximum of the SCL curve stands out sharply, suggesting a clear-cut threshold that separates a group of more similar molecules from groups of less similar molecules. On the other hand, missing this *t*_*α*_ just slightly might lead to a less effective separation of molecule groups. By comparison, the local *ACC*(*t*) maximum in large datasets might be considered more robust, that is, slightly missing *t*_*α*_ will not cause such a sudden change in the separation between groups of related molecules.

The observed differences in the characteristics of the *ACC*(*t*) and *EN*(*t*) functions are influenced by the applied similarity measure between the molecules. In this experimental framework, we use only one type of fingerprint and similarity measure, i.e. the ECFP_4 fingerprint and Tanimoto similarity-coefficient, the location of *t*_*α*_ and the characteristics of the *ACC*(*t*) and *EN*(*t*) functions are dependent on the similarity measure at hand. This effect is demonstrated by several examples in the Supporting Material (see: Additional file 1: Fig. S1, Additional file 2: Fig. S2, Additional file 3: Fig. S3, Additional file 4: Fig. S4, Additional file 5: Fig. S5, Additional file 6: Fig. S6, Additional file 7: Fig. S7).

The emergence of a robust local maximum of the *ACC*(*t*) is also demonstrated on an additional much larger dataset extracted from the ChEMBL 20 database [42] (downloaded on 04/24/2015). This dataset contains more than one million molecules and it was analyzed by the Snap library [43]. The threshold associated with that local maximum is also well-aligned with the sudden change in the slope of the *EN*(*t*) function. These results are included in the Supporting Material (see: Additional file 8: Fig. S8).

### Clustering performance as function of the similarity threshold

Analyzing the clustering performance as function of the similarity threshold requires that certain factors are kept invariant through the clustering process. The similarity matrices were generated with the ECFP_4 fingerprint algorithm and the Tanimoto similarity measure. While different choices can be made in selecting the applied clustering algorithm and performance measure, our goal was to choose a reliable clustering algorithm and a widely used performance measure. As detailed in *Datasetsand Methods*, we used the InfoMap algorithm on a 200+ core computing cluster, and *sensitivity* and *specificity* to characterize clustering performance.

The algorithm is able to detect the number of clusters automatically thus alleviating the need to input this number *a priori*. For reasons related to storing the similarity matrices, the similarity threshold evaluation began at *t* = 0.30 in the case of the WOMBAT and MLSMR datasets, to assure that the produced networks can be stored and processed by the available computational tools.

**Fig. 6:**
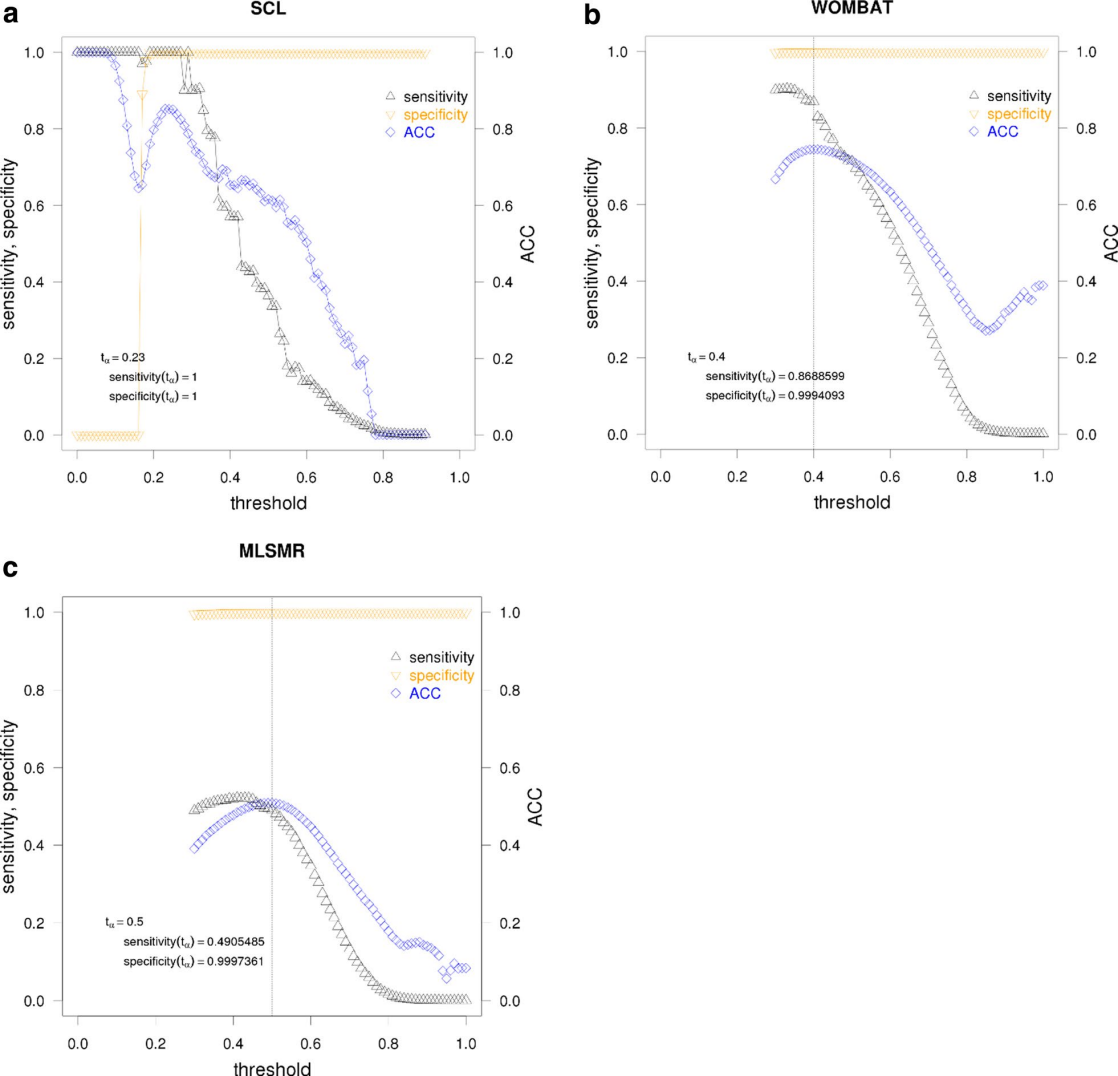
Clustering performance in the function of the similarity threshold. On each figure shown are the sensitivity and specificity values associated with the determined *t*_*α*_, i.e. the ‘obvious’ local maximum to choose. *Dashed vertical line* indicates the location of *t*_*α*_ on the x-axis. **a** In the case of the SCL dataset both sensitivity and specificity values meet the ideal value of 1 over a range of similarity thresholds (0.19 ≤ *t* ≤ 0.27 and at *t* = 0.23). Please note that above *t* = 0.91 the similarity network only consists of singletons, therefore the respective experimental points are not displayed on the graph. **b** In the case of the WOMBAT dataset the value of sensitivity and specificity associated with *t*_*α*_ = 0.40 are 0.8689 and 0.9994, respectively. The deviation between these values and their observed maximum is acceptable. **c** In the case of the PubChem MLSMR dataset the sensitivity and specificity associated with *t*_*α*_ = 0.50 are 0.4905 and 0.9997, respectively. The deviation between these values and their observed maximum is acceptable

As seen in Fig. 6, the clustering performance is as dependent on the selected similarity threshold as on the *ACC*. The *specificity* of the clustering is close to the maximum over the majority of the range of the selected threshold, which means that molecules that are *not* supposed to be clustered together are, indeed, *not* clustered together given a reference or pseudo-reference clustering. Thus, the resultant clusters can be thought of as being homogeneous. On the other hand, the ideal situation, characterized by *sensitivity* = *1* and *specificity* = 1 is only observed for the SCL dataset. This may be indicative of internal consistency within a data set, i.e. less heterogeneity and more self-similarity among molecules within the clustered set.

While the observed maximum *sensitivity* is near maximal in the case of WOMBAT dataset, the same parameter has a rather low value for MLSMR, reaching its maximum at *sensitivity* = 0.5223. Analyzing the causes of this difference is beyond the scope of the current study. Certain hints are unveiled by the *ACC*, as discussed in the following subsections. Furthermore, the number of singletons accounting for such a difference in *sensitivity* values can be ruled out, as shown in Additional file 9: Fig. S9, Additional file 10: Fig. S10. For the sake of comparison, additional information is provided for the SCL dataset in Additional file 11: Fig. S11. A complete analysis would require computing the missing range of threshold values for the WOMBAT and the MLSMR datasets, which is at the moment a challenge.

### Relation of clustering performance and the observed maximum of ACC versus similarity threshold function

As shown above, the selection of the similarity threshold has a critical effect on the topology of the resultant similarity network which, in turn, substantially affects clustering performance. Evaluating the resultant clustering is a rather difficult step, which typically involves the use of a reference clustering set. Such reference clustering sets at large scale are scarce, if available at all. However, we presumed that the *ACC*(*t*) function could provide insight in the quality of the clustering even if no reference dataset exists. Therefore, we analyzed whether the *ACC*(*t*) function can suggest a threshold within a given framework (i.e. a similarity measure and a clustering algorithm) that might lead to a reasonable clustering, even if a reference clustering set was not available. We intended to analyze what clues are provided by the *ACC*(*t*) that could be used to describe the structure of the underlying data, and to inform us whether the similarity measure of choice can be improved for the dataset at hand. While some of these objectives might sound trivial in the case of small datasets, they can be relevant for datasets in the hundreds of thousands of molecules range.

The first and probably most apparent feature of the *ACC*(*t*) functions in this study is the presence of a local maximum, which is often ’obvious’, i.e. a *t*_*α*_ that is clearly distinguishable from smaller local maxima. This is *not* the local maximum at *t* = 0, which yields an *ACC* of 1. In general, this *t*_*α*_ might be characterized as robust, because its peak spans a larger range of the similarity threshold than any other peak. In some cases, as shown in the Supporting Material [Additional file 3: Fig. S3(a)], the peak enclosing *t*_*α*_ might contain other, minor peaks belonging to other local maxima, but it is still obvious that they are part of a larger peak, which encloses *t*_*α*_.

Although threshold values below 0.30 are not evaluated for the WOMBAT and MLSMR datasets, a robust local maximum is observed, spanning a larger range of similarity threshold values than any other local maximum. Should a local maximum appear below 0.30, the peak associated to that local maximum could only span a smaller range of threshold values than the peak associated to the visible *t*_*α*_. In the case of SCL dataset *t*_*α*_ coincides with the threshold where an ideal clustering performance of *sensitivity* = *1* and *specificity* = *1* is achieved (see: Fig. 6a). For both the WOMBAT an MLSMR datasets, selecting the threshold at *t*_*α*_ would yield a clustering performance near an optimal value, with very little differences (see: Fig. 6b, c). The word “optimal” is used here to reflect the fact that only a part of the entire threshold range is available for analysis. It is possible, however, that in the case of the WOMBAT dataset the visible *t*_*α*_ might also be the *t*_*α*_ for the entire threshold range. This assumption is based on the high value of the observed *ACC* of *t*_*α*_.

As mentioned earlier, the manual analysis of datasets in the size of hundreds of thousands of molecules is infeasible. In order to further support the value in identifying *t*_*α*_ of the *ACC*(*t*) function illustrative examples are provided in the Supporting Material (see: Additional file 12, Additional file 13, Additional file 14, Additional file 15). These examples contrast the quality of clustering in the case of the WOMBAT and PubChem MLSMR datasets by setting the threshold to *t*_*α*_ of the *ACC*(*t*) function as compared to setting it on the basis of an in-house practice. This in-house practice favors the threshold associated with the highest number of observed clusters, excluding singletons. Although the corresponding clusters are equally cohesive, the clusters obtained at *t*_*α*_ contain more molecules of the kind. This means that the clusters are split at the threshold associated with the highest number of clusters (singletons excluded). Although only one example is provided for both datasets, this trend is clear when considering the sensitivity and specificity values in the function of the similarity threshold, as described above.

For SCL, the *ACC*(*t*_*α*_) value is above 0.8, which suggests a high neighbors connectivity in the similarity network. A similar observation can be made for the WOMBAT dataset, because the visible *ACC*(*t*_*α*_) is a little below 0.8. On the other hand, *ACC*(*t*_*α*_) is quite low for the MLSMR dataset. Thus, the SCL and WOMBAT datasets have groups of similar and less similar objects better separated at *t*_*α*_, which might offer valuable information regarding the diversity of the underlying datasets, given the applied similarity measure.

The intent of the MLSMR was to serve as a “diversity” library, which suggests that deliberate steps were taken to ensure a high number of dis-similar chemicals were incorporated. By contrast, WOMBAT is comprised of a number of literature-extracted sets; and more often than not, each paper consists of a low number of scaffold-based analog series (usually 1, but rarely above 5 such series). This is consistent with the *ACC*(*t*_*α*_) trends noted above.

Analyzing the same datasets but using different similarity measures, shown in the Supporting Material [Additional file 16: Fig. S12(a)], may lead to a different conclusion, namely that a particular similarity measure is more appropriate for one dataset compared to another. This is a known challenge in the field of cheminformatics, as fingerprints determine the resolution of defining similarity between pairs of molecules. Thus, analyzing the *ACC*(*t*) function of a dataset might be of value to decide which fingerprint is more appropriate in the light of a given investigative objective.

In the *Supporting Material* further insight is provided in terms of relating the clustering performance to the similarity network topology (see: “Appendix: First and second order derivatives of the number of edges vs. threshold functions”). A detailed discussion of these findings is beyond the scope of this paper.

In summary, we were able to demonstrate the emergence of an obvious local maximum of the *ACC*(*t*) function associated with *t*_*α*_ in the case of all datasets. The three datasets evaluated above share one important feature: Namely, they contain molecules that can be part of various SAR series. Despite the differences in the diversity of the SCL, WOMBAT and PubCHEM MLSMR sets and the value of *ACC* observed at *t*_*α*_ it holds true that the observed best clustering performance is well aligned with *t*_*α*_. These results support the feasibility of extending and generalizing our original similarity threshold selection approach [4] for large datasets.

Although WOMBAT and MLSMR are in the range of 10^5^ molecules, computing the *ACC*(*t*) function for larger molecular datasets is possible. We have computed this function for the ChEMBL 20 dataset that contains more than 1.2 × 10^6^ molecules (see: Additional file 8: Fig. S8). The emergence of an obvious local maximum of the *ACC*(*t*) function was indeed observed. Considering that the computation of this function can be adopted to a parallel-computing environment, we expect that computing the *ACC*(*t*) function should not be difficult for even larger datasets.

Besides computing the *ACC*(*t*) function, the other limiting step in clustering larger molecular datasets is the clustering algorithm itself. InfoMap can be substituted by another clustering algorithm, the similarity threshold selection method allows for it. Accordingly, the parallelization of the InfoMap algorithm or the use of an alternative method can further push the size of manageable molecular datasets.

## Conclusions

In this study we proposed a systematic method and an objective measure to select the threshold to be applied on a similarity matrix of molecules for network-based clustering. Finding an appropriate similarity cut-off value affects clustering performance and results, as demonstrated by analyzing three different datasets. We provide a clustering framework suitable to perform clustering and evaluate clustering performance on a large dataset. Monitoring the *ACC* as function of the cut-off value can reveal a threshold that improves the likelihood of obtaining a reasonable clustering performance when a network-based clustering algorithm was deployed. Moreover, we demonstrate that the average clustering coefficient can provide insight regarding the diversity of the dataset at hand and how the choice of the fingerprint algorithm can be improved. This latter property has substantial influence on clustering outcome. In the beginning of the era of *Big Data* it is of great importance to devise algorithms that can improve the quality of clustering for large datasets when human quality control would become intractable or unreliable.

## Outlook

Considering that the size of chemical databases can be expected to increase substantially, and given that the computational costs of computing the *ACC* for a network will increase, it may be of interest to explore the use of heuristics based methods to approximate the *ACC*. An alternative method of detecting important changes in network topology is the approximation of the first and second order derivatives of the number of edges versus threshold function. Furthermore, it could be of interest to apply an asymmetric similarity measure, e.g. Tversky [44] as opposed to the Tanimoto similarity-coefficient. This approach could lead to directed weighted and directed unweighted networks that might reveal further insight among molecular structures.

## Appendix: First and second order derivatives of the number of edges versus threshold functions

Let *f*(*x*) denote the function of the number of edges in the similarity network in the function of the selected similarity threshold *x*. In order to investigate whether the best clustering performance is aligned with the first and second order derivatives of *f*(*x*) we approximated them with the help of numerical differentiation [45]. We applied three different methods to compute the first order derivative, namely the so-called forward, backward and central difference as defined in Eqs. 6–8, respectively [46].

6 $$f^{{\prime }} \left( x \right) = \frac{{f\left( {x + d} \right) - f\left( x \right)}}{d}$$

7 $$f^{\prime } \left( x \right) = \frac{{f\left( x \right) - f\left( {x - d} \right)}}{d}$$

8 $$f^{\prime } \left( x \right) = \frac{{f\left( {x + d} \right) - f\left( {x - d} \right)}}{2d}$$

The second order derivative was computed according to Eq. 9.

9 $$f^{\prime \prime } \left( x \right) = \frac{{f\left( {x + d} \right) - 2f\left( x \right) + f\left( {x - d} \right)}}{{d^{2} }}$$

In the above equations (Eqs. 6–9) *d* denotes the similarity threshold difference. It was selected to be 0.01 in the numerical differentiations as it is the same value as the increment of similarity threshold applied in all experiment.

The first and second order derivatives of *f*(*x*) in the case of the SCL, WOMBAT and PubChem MLSMR datasets are shown on Additional file 17: Fig. S13, Additional file 18: Fig. S14, Additional file 19: Fig. S15. In the case of the WOMBAT and PubChem MLSMR datasets the similarity threshold (*t*_*γ*_) associated with the observed best clustering performance is represented by a vertical dotted line (see: Additional file 18: Fig. S14, Additional file 19: S15). The value of *t*_*γ*_ was determined by identifying where the sum of sensitivity and specificity is maximal. In the case of the SCL dataset the possible best clustering performance is achieved at multiple values of the similarity threshold therefore no single *t*_*γ*_ and the corresponding vertical line is indicated on the graph (see: Additional file 17: Fig. S13).

In the case of the WOMBAT and PubChem MLSMR data sets the second order derivative of *f*(*x*) has a local maximum that is aligned with *t*_*γ*_. Furthermore, in the case of the PubChem MLSMR dataset the first order derivative of *f*(*x*) computed via the backward difference has a local minimum aligned with *t*_*γ*_.

In the case of the SCL dataset both the first and second order derivatives produce multiple local maxima and minima at thresholds associated with the potential best clustering outcome. It should be noted that in the case of the first derivative the aforementioned observation holds true, regardless of the selected difference computation method. Furthermore, the second order derivative also produces a zero value at one of the thresholds associated with the best clustering outcome.

## Additional files

10.1186/s13321-016-0127-5 Topological features of the similarity network created by using the SCL dataset, ChemAxon 1024 bit hashed fingerprint and Tanimoto similarity-coefficient. Similarity threshold is increased in increments of 0.01 from 0.00 to 1.00.
10.1186/s13321-016-0127-5 Topological features of the similarity network created by using the SCL dataset, ChemAxon 2048 bit hashed fingerprint and Tanimoto similarity-coefficient. Similarity threshold is increased in increments of 0.01 from 0.00 to 1.00.
10.1186/s13321-016-0127-5 Topological features of the similarity network created by using the SCL dataset, ChemAxon 4096 bit hashed fingerprint and Tanimoto similarity-coefficient. Similarity threshold is increased in increments of 0.01 from 0.00 to 1.00.
10.1186/s13321-016-0127-5 Topological features of the similarity network created by using the SCL dataset, MACCS fingerprint and Tanimoto similarity-coefficient. Similarity threshold is increased in increments of 0.01 from 0.00 to 1.00.
10.1186/s13321-016-0127-5 Topological features of the similarity network created by using the SCL dataset, ECFP_4 fingerprint and Tanimoto similarity-coefficient. Similarity threshold is increased in increments of 0.01 from 0.00 to 1.00.
10.1186/s13321-016-0127-5 Topological features of the similarity network created by using the SCL dataset, ECFP_8 fingerprint and Tanimoto similarity-coefficient. Similarity threshold is increased in increments of 0.01 from 0.00 to 1.00.
10.1186/s13321-016-0127-5 Topological features of the similarity network created by using the SCL dataset, ECFP_12 fingerprint and Tanimoto similarity-coefficient. Similarity threshold is increased in increments of 0.01 from 0.00 to 1.00.
10.1186/s13321-016-0127-5 Analysis of the ChEMBL 20 dataset. Molecular structures were extracted from the ChEMBL 20 version (downloaded on 04/24/2015). The structures were subject to an identical standardization procedure as described in the case of the three other datasets, i.e. the SCL, WOMBAT and MLSMR PubChem datasets. Standardization was performed using ChemAxon’s JChem *standardize* utility (version 15.8.10.0). The ChEMBL 20 dataset comprises 1,256,876 unique molecules that have a MW ≤ 700 and atomcount ≤ 80. In order to generate the similarity networks in the function of the similarity threshold ECFP fingerprints were generated for the molecules with a diameter of 4. Similarity of the molecules was quantified by the Tanimoto-similarity measure. The range of applied similarity threshold *t* is 0.30 ≤ *t* ≤ 1.00 and *t* was incremented in steps of 0.01. (a) The number of edges in the similarity network in the function of the similarity threshold. (b) The average clustering coefficient (ACC) in the function of the applied similarity threshold. The obvious local maximum of the ACC vs. threshold curve is at threshold *t*_*α*_ = 0.48. The value of the associated ACC(*t*_*α*_) is 0.5979.
10.1186/s13321-016-0127-5 Number of clusters and singletons in the function of the selected threshold, WOMBAT dataset. Fingerprint: ECFP_4, similarity measure: Tanimoto similarity-coefficient, clustering algorithm: InfoMap, similarity threshold *t* incremented in steps of 0.01 in the range of 0.30 ≤ *t* ≤ 1.00. (a) Number of clusters excluding singletons. The highest number of clusters, 18,120, is observed at *t* = 0.72. (b) Number of clusters including singletons. (c) Number of singletons.
10.1186/s13321-016-0127-5 Number of clusters and singletons in the function of the selected threshold, MLSMR dataset. Fingerprint: ECFP_4, similarity measure: Tanimoto similarity-coefficient, clustering algorithm: InfoMap, similarity threshold *t* incremented in steps of 0.01 in the range of 0.30 ≤ *t* ≤ 1.00. (a) Number of clusters excluding singletons. The highest number of clusters, 40,244, is observed at *t* = 0.68. (b) Number of clusters including singletons. (c) Number of singletons.
10.1186/s13321-016-0127-5 Number of clusters and singletons in the function of the selected threshold, SCL dataset. Fingerprint: ECFP_4, similarity measure: Tanimoto similarity-coefficient, clustering algorithm: InfoMap, similarity threshold *t* incremented in steps of 0.01 in the range of 0.00 ≤ *t* ≤ 0.91. Note, that above *t* = 0.91 the similarity network only consists of singletons, therefore the respective experimental points are not displayed on the graph. (a) Number of clusters excluding singletons. (b) Number of clusters including singletons. (c) Number of singletons.
10.1186/s13321-016-0127-5 Illustrative cluster of WOMBAT dataset at threshold = 0.40. File name: wombat_nm17_cid_1178_t_alpha_0.40_pub.pdf . Shown are the molecules of cluster 1178 of WOMBAT dataset produced at the obvious local maximum of the ACC vs. threshold curve at threshold *t*_*α*_ = 0.40. PDF generated by ChemAxon’s *mview* utility.
10.1186/s13321-016-0127-5 Illustrative cluster of WOMBAT dataset at threshold = 0.72. File name: wombat_nm17_cid_505_t_0.72_pub.pdf . Shown are the molecules of cluster 505 of WOMBAT dataset produced at threshold *t* = 0.72 associated with the highest number of clusters (singletons excluded). PDF generated by ChemAxon’s *mview* utility.
10.1186/s13321-016-0127-5 Illustrative cluster of PubChem MLSMR dataset at the threshold = 0.50. File name: mlsmr_nm16_t_alpha_0.50_cid_674_pub.pdf. Shown are the molecules of cluster 674 of PubChem MLSMR dataset produced at the obvious local maximum of the ACC vs. threshold curve at threshold *t*_*α*_ = 0.50. PDF generated by ChemAxon’s *mview* utility.
10.1186/s13321-016-0127-5 Illustrative cluster of PubChem MLSMR dataset at the threshold = 0.68. File name: mlsmr_nm16_t_0.68_cid_100_pub.pdf . Shown are the molecules of cluster 100 of PubChem MLSMR dataset produced at threshold *t* = 0.68 associated with the highest number of clusters (singletons excluded). PDF generated by ChemAxon’s *mview* utility.
10.1186/s13321-016-0127-5 The effect of the applied fingerprint on the network topology in the case of SCL dataset. Tanimoto similarity threshold was incremented by steps of 0.01 in the range of 0 to 1. The choice of molecular fingerpint generating method has a profound effect on both the *ACC*(*t*) and *EN*(*t*) functions.
10.1186/s13321-016-0127-5 First and second order derivatives of the number of edges vs. threshold function in the case of the SCL dataset. The aforementioned function is denoted by *f*(*x*), and it’s first and second order derivatives by *f′*(*x*) and *f″*(*x*), respectively. The derivatives were approximated by numerical differentiation. First order derivatives: (a) using the forward difference, (b) using the backward difference, (c) using the central difference. (d) Second order derivative.
10.1186/s13321-016-0127-5 First and second order derivatives of the number of edges vs. threshold function in the case of the WOMBAT dataset. The aforementioned function is denoted by *f*(*x*), and it’s first and second order derivatives by *f′*(*x*) and *f″*(*x*), respectively. The derivatives were approximated by numerical differentiation. The vertical line at threshold *t*_*γ*_ denotes the threshold associated with the observed best clustering performance. First order derivatives: (a) using the forward difference, (b) using the backward difference, (c) using the central difference. (d) Second order derivative.
10.1186/s13321-016-0127-5 First and second order derivatives of the number of edges vs. threshold function in the case of the PubChem MLSMR dataset. The aforementioned function is denoted by *f*(*x*), and it’s first and second order derivatives by *f′*(*x*) and *f″*(*x*), respectively. The derivatives were approximated by numerical differentiation. The vertical line at threshold *t*_*γ*_ denotes the threshold associated with the observed best clustering performance. First order derivatives: (a) using the forward difference, (b) using the backward difference, (c) using the central difference. (d) Second order derivative.

## References

[1] Palla G, Derényi I, Farkas I, Vicsek T (2005). Uncovering the overlapping community structure of complex networks in nature and society. Nature.

[2] Derényi I, Palla G, Vicsek T (2005). Clique percolation in random networks. Phys Rev Lett.

[3] Adamcsek B, Palla G, Farkas IJ, Derényi I, Vicsek T (2006). CFinder: locating cliques and overlapping modules in biological networks. Bioinformatics.

[4] Zahoránszky LA, Katona GY, Hári P, Málnási-Csizmadia A, Zweig KA, Zahoránszky-Köhalmi G (2009). Breaking the hierarchy—a new cluster selection mechanism for hierarchical clustering methods. Algorithms Mol Biol.

[5] Rosvall M, Bergstrom CT (2008). Maps of random walks on complex networks reveal community structure. Proc Natl Acad Sci USA.

[6] Girvan M, Newman MEJ (2002). Community structure in social and biological networks. Proc Natl Acad Sci USA.

[7] Augustson JG, Minker J (1970). An analysis of some graph theoretical cluster techniques. J ACM.

[8] Saito S, Hirokawa T, Horimoto K (2011). Discovery of chemical compound groups with common structures by a network analysis approach (affinity prediction method). J Chem Inf Model.

[9] Tanaka N, Ohno K, Niimi T, Moritomo A, Mori K, Orita M (2009). Small-world phenomena in chemical library networks: application to fragment-based drug discovery. J Chem Inf Model.

[10] Watts DJ, Strogatz SH (1998). Collective dynamics of ‘small-world’ networks. Nature.

[11] Wawer M, Peltason L, Weskamp N, Teckentrup A, Bajorath J (2008). Structure-activity relationship anatomy by network-like similarity graphs and local structure-activity relationship indices. J Med Chem.

[12] Software S: MACCS strutural keys. San Ramon, CA

[13] Serrano MA, Boguñá M, Vespignani A (2009). Extracting the multiscale backbone of complex weighted networks. Proc Natl Acad Sci USA.

[14] Barupal DK, Haldiya PK, Wohlgemuth G, Kind T, Kothari SL, Pinkerton KE, Fiehn O (2012). MetaMapp: mapping and visualizing metabolomic data by integrating information from biochemical pathways and chemical and mass spectral similarity. BMC Bioinformatics.

[15] Horvát E-Á, Zhang JD, Uhlmann S, Sahin Ö, Zweig KA (2013). A network-based method to assess the statistical significance of mild co-regulation effects. PLoS One.

[16] Weininger D (1988). SMILES, a chemical language and information system. 1. Introduction to methodology and encoding rules. J Chem Inf Model.

[17] Albany Molecular Research Inc. http://www.amriglobal.com/

[18] Irwin JJ, Shoichet BK (2004). ZINC–a free database of commercially available compounds for virtual screening. J Chem Inf Model.

[19] Olah M, Rad R, Ostopovici L, Bora A, Hadaruga N, Hadaruga D, Moldovan R, Fulias A, Mracec M, Oprea TI, Schreiber SL, Kapoor TM, Wess G (2007). WOMBAT and WOMBAT-PK: bioactivity databases for lead and drug discovery. Chemical biology: from small molecules to systems biology and drug design.

[20] PML Program, “Program, PubChem Molecular Libraries”

[21] Langdon SR, Brown N, Blagg J (2011). Scaffold diversity of exemplified medicinal chemistry space. J Chem Inf Model.

[22] Bemis GW, Murcko MA (1996). The properties of known drugs. 1. Molecular frameworks. J Med Chem.

[23] Nilakantan R, Bauman N, Dixon JS, Venkataraghavan R (1987). Topological torsion: a new molecular descriptor for SAR applications. Comparison with other descriptors. J Chem Inf Model.

[24] Bolton EE, Wang Y, Thiessen PA, Bryant SH (2010). PubChem: integrated platform of small molecules and biological activities. Annu Rep Comput Chem.

[25] ChemAxon Ltd., Chemical Hashed Fingerprints. http://www.chemaxon.com/jchem/doc/user/fingerprint.html

[26] Maldonado AG, Doucet JP, Petitjean M, Fan B-T (2006). Molecular similarity and diversity in chemoinformatics: from theory to applications. Mol Divers.

[27] Leach AR (2001). Molecular modelling: principles and applications.

[28] Brown RD, Martin YC (1996). Use of structure-activity data to compare structure-based clustering methods and descriptors for use in compound selection. J Chem Inf Model.

[29] Willett P, Barnard JM, Downs GM (1998). Chemical similarity searching. J Chem Inf Model.

[30] O’Boyle NM, Banck M, James CA, Morley C, Vandermeersch T, Hutchison GR (2011). Open Babel: an open chemical toolbox. J Cheminform.

[31] Ehrman JR (1968). ‘Logical’ arithmetic on computers with two’s complement binary arithmetic. Commun ACM.

[32] Morgan HL (1965). The generation of a unique machine description for chemical structures—a technique developed at chemical abstracts service. J Chem Doc.

[33] Rogers D, Hahn M (2010). Extended-connectivity fingerprints. J Chem Inf Model.

[34] Weininger D, Weininger A, Weininger JL (1989). SMILES. 2. Algorithm for generation of unique SMILES notation. J Chem Inf Model.

[35] Tanimoto TT (1957) IBM internal report

[36] Gardiner EJ, Gillet VJ, Willett P, Cosgrove DA (2007). Representing clusters using a maximum common edge substructure algorithm applied to reduced graphs and molecular graphs. J Chem Inf Model.

[37] Raymond JW (2002). RASCAL: calculation of graph similarity using maximum common edge subgraphs. Comput J.

[38] Wilkens SJ, Janes J, Su AI (2005). HierS: hierarchical scaffold clustering using topological chemical graphs. J Med Chem.

[39] Yang JJ, “Google Code open source project, unm-biocomp-hscaf, Java library for HierS chemical scaffolds”

[40] Fortunato S (2010). Community detection in graphs. Phys Rep.

[41] Altman DG, Bland JM (1994). Statistics notes: diagnostic tests 1: sensitivity and specificity. BMJ.

[42] Bento AP, Gaulton A, Hersey A, Bellis LJ, Chambers J, Davies M, Krüger FA, Light Y, Mak L, McGlinchey S, Nowotka M, Papadatos G, Santos R, Overington JP (2014). The ChEMBL bioactivity database: an update. Nucleic Acids Res.

[43] Leskovec J, Sosič R (2014) {SNAP}: a general purpose network analysis and graph mining library in {C++} 10.1145/2898361 PMC5361061 28344853

[44] Tversky A (1977). Features of similarity. Psychol Rev.

[45] Analysis suggested by Reviewer #1

[46] Kiusalaas J (2005). Numerical methods in engineering with Matlab.

