# Erratum to: Impact of similarity threshold on the topology of molecular similarity networks and clustering outcomes

**DOI:** 10.1186/s13321-016-0140-8

**Published:** 2016-05-20

**Authors:** Gergely Zahoránszky-Kőhalmi, Cristian G. Bologa, Oleg Ursu, Tudor I. Oprea

**Affiliations:** Translational Informatics Division, University of New Mexico School of Medicine, MSC09 5025, Albuquerque, NM 87131 USA

## Erratum to: J Cheminform (2016) 8:16 DOI 10.1186/s13321-016-0127-5

After publication of this study [1], the authors noticed that Oleg Ursu was not listed among the authors. This error has now been corrected, and the full list of authors contributing to this work is included.

The Author contributions statement has also been corrected in order to reflect this change. The correct information is given below:

The section “Reference clustering data sets” contains the description of an algorithm for generating reference data based on idea, algorithm and Java implementation by Oleg Ursu, PhD. The first version of this algorithm was presented at the ChemAxon User Group Meeting in San Diego, 2009 (https://www.chemaxon.com/library/natural-clustering-an-approximate-mces-algorithm-used-on-wombat/). Specifically Dr. Ursu had the idea to combine approximate clique detection with exact clique detection in order to significantly improve MCES algorithm computational time. Furthermore, this algorithm uses a heuristic-based approach: by starting with the hierarchical scaffold algorithm (HierS; designed and implemented in-house by Jeremy Yang), Dr Ursu’s method prunes the cluster search space using HierS heuristics and then applies approximate or exact clique detection algorithms based on molecule size. The choice of exact versus approximate clique detection was also coded by Dr. Ursu, based on empirical observations of algorithm run-times for various molecule sizes.

We therefore consider Dr. Ursu’s contribution to be substantial, since without his method the “pseudo-reference set” would have not been available. Dr. Ursu’s work has not been made available in the public domain prior to this paper.
