# Supplementary material for: Impact of similarity threshold on the topology of molecular similarity networks and clustering outcomes
Source: J Cheminform. 2016 Mar 30;8:16. doi: 10.1186/s13321-016-0127-5 (PMC4812625; doi:10.1186/s13321-016-0127-5)

Additional file 4: Figure S4: Topological features of the similarity network created by using the SCL dataset, MACCS fingerprint and Tanimoto similarity-coefficient. Similarity threshold is increased in increments of 0.01 from 0.00 to 1.00.


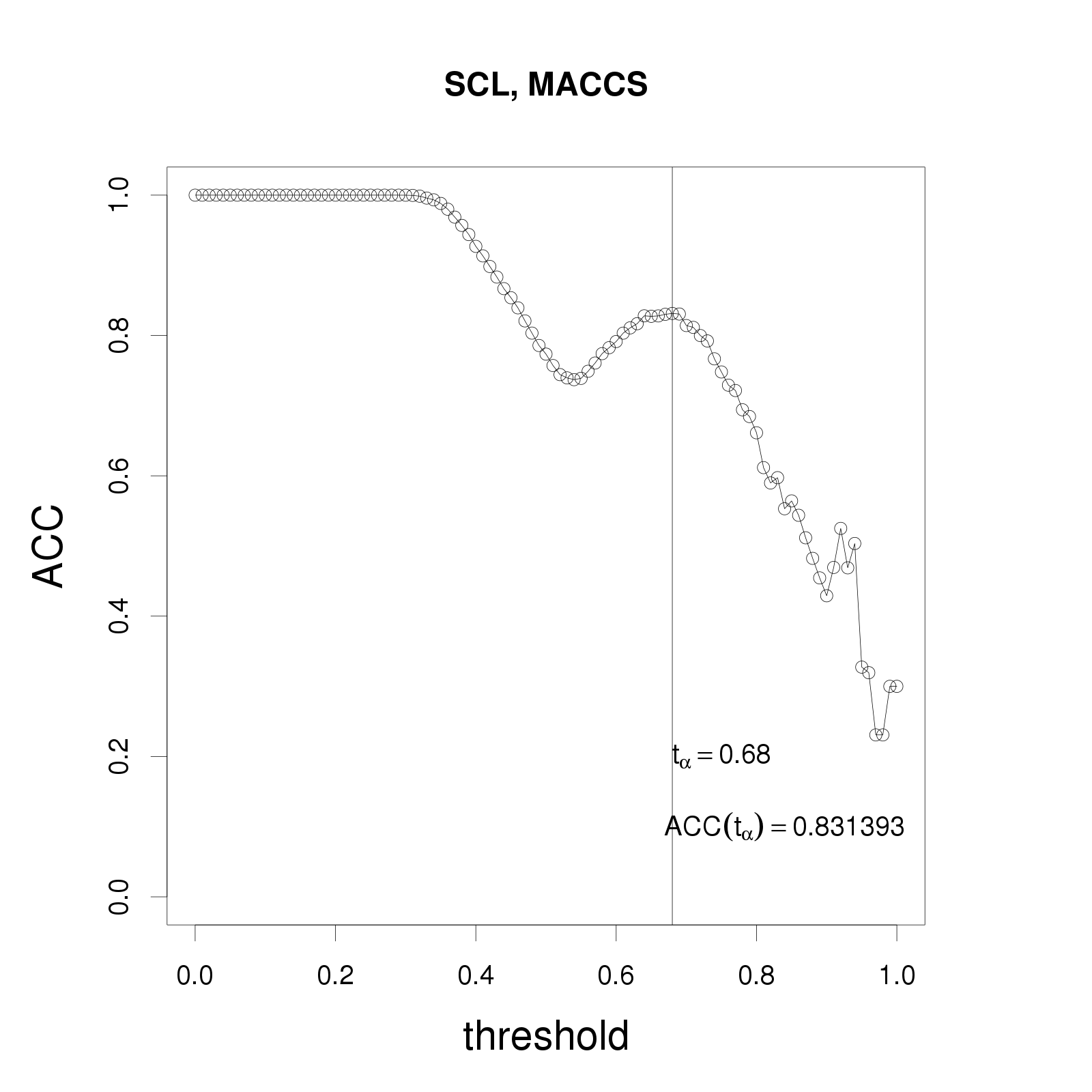


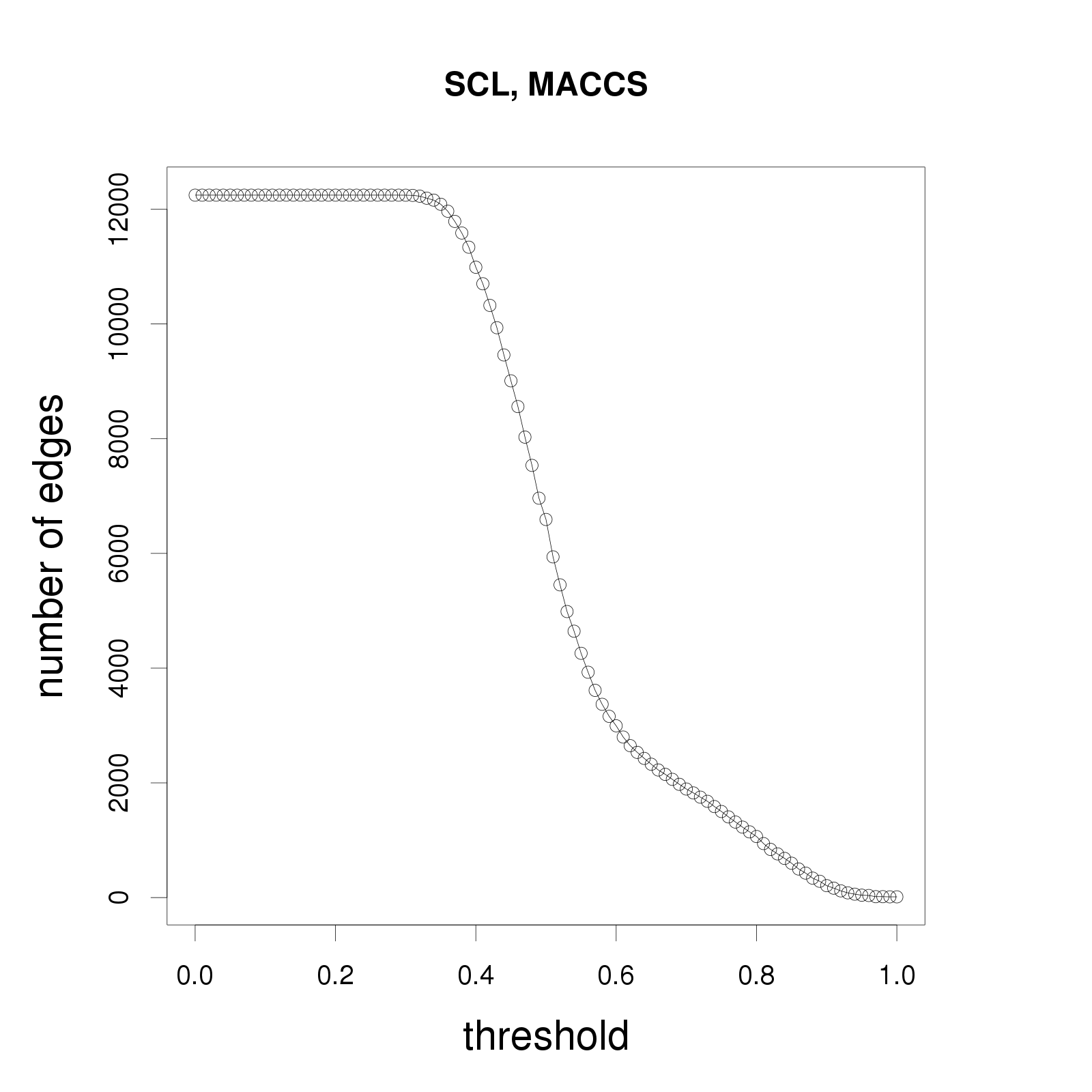

Supplement: Supplementary file 4 — 10.1186/s13321-016-0127-5 Topological features of the similarity network created by using the SCL dataset, MACCS fingerprint and Tanimoto similarity-coefficient. Similarity threshold is increased in increments of 0.01 from 0.00 to 1.00. [file 13321_2016_127_MOESM4_ESM.docx]
