# Supplementary material for: Impact of similarity threshold on the topology of molecular similarity networks and clustering outcomes
Source: J Cheminform. 2016 Mar 30;8:16. doi: 10.1186/s13321-016-0127-5 (PMC4812625; doi:10.1186/s13321-016-0127-5)

Additional file 8: Figure S8: Analysis of the **ChEMBL** 20 dataset. Molecular structures were extracted from the ChEMBL 20 version (downloaded on 04/24/2015). The structures were subject to an identical standardization procedure as described in the case of the three other datasets, i.e., the SCL, WOMBAT and MLSMR PubChem datasets. Standardization was performed using ChemAxon’s JChem *standardize* utility (version 15.8.10.0). The ChEMBL 20 dataset comprises 1256876 unique molecules that have a MW <= 700 and atomcount <= 80. In order to generate the similarity networks in the function of the similarity threshold ECFP fingerprints were generated for the molecules with a diameter of 4. Similarity of the molecules was quantified by the Tanimoto-similarity measure. The range of applied similarity threshold is 0.30 <= threshold <= 1.00 and the threshold was incremented in steps of 0.01. (a) The number of edges in the similarity network in the function of the similarity threshold. (b) The average clustering coefficient (ACC) in the function of the applied similarity threshold. The obvious local maximum of the ACC vs. threshold curve is at threshold *t_α_* = 0.48. The value of the associated ACC(*t_α_*) is 0.5979.


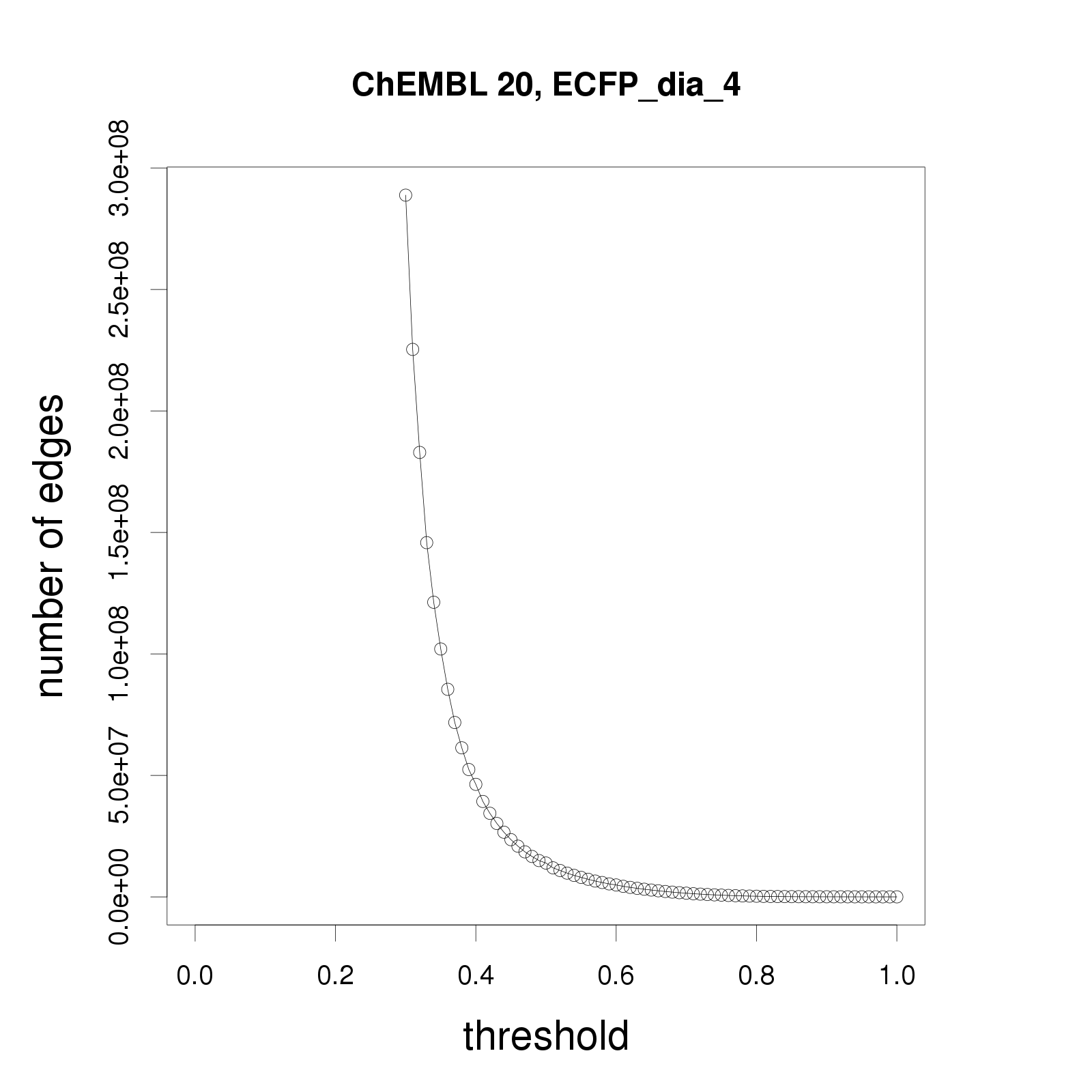


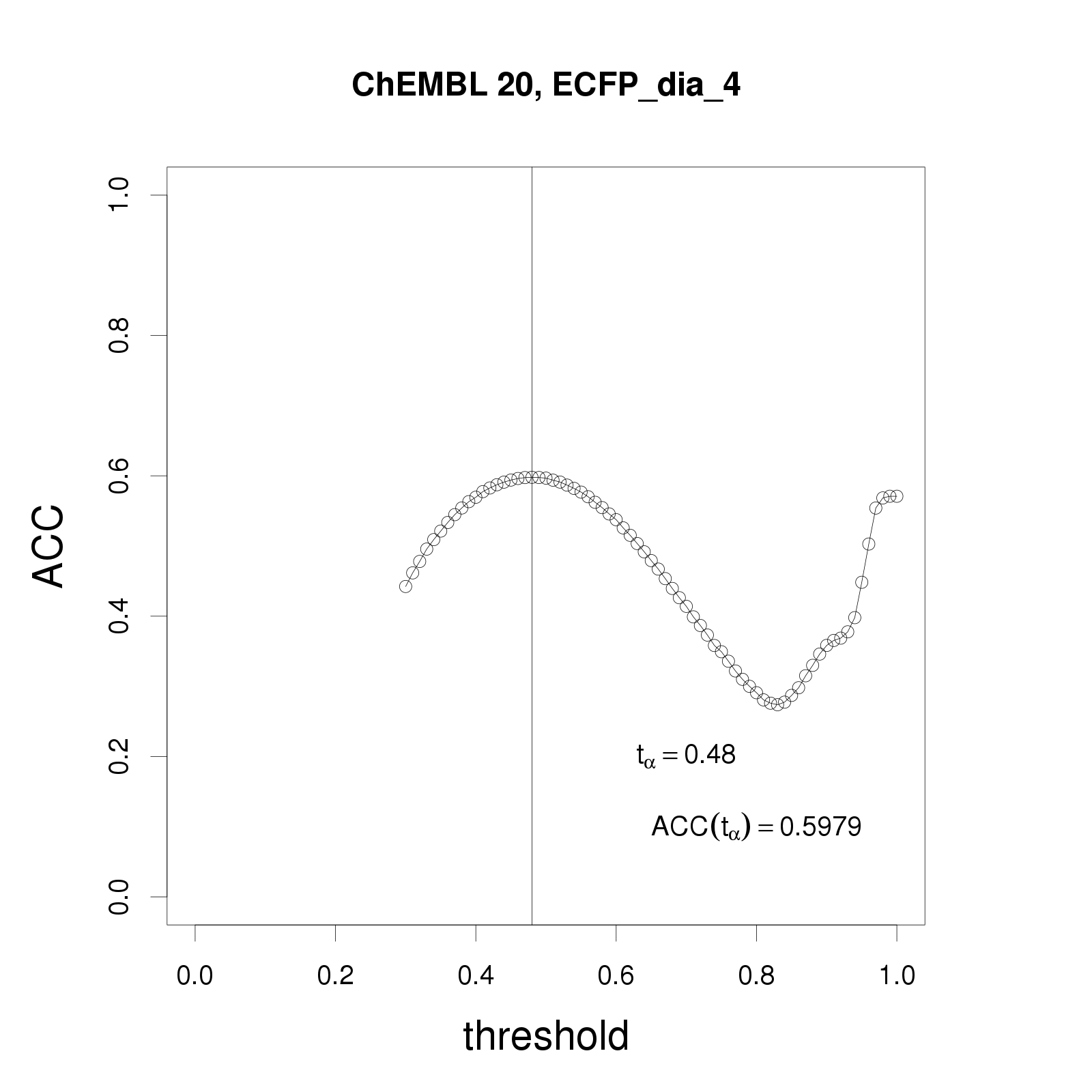

Supplement: Supplementary file 8 — 10.1186/s13321-016-0127-5 Analysis of the ChEMBL 20 dataset. Molecular structures were extracted from the ChEMBL 20 version (downloaded on 04/24/2015). The structures were subject to an identical standardization procedure as described in the case of the three other datasets, i.e. the SCL, WOMBAT and MLSMR PubChem datasets. Standardization was performed using ChemAxon’s JChem standardize utility (version 15.8.10.0). The ChEMBL 20 dataset comprises 1,256,876 unique molecules that have a MW ≤ 700 and atomcount ≤ 80. In order to generate the similarity networks in the function of the similarity threshold ECFP fingerprints were generated for the molecules with a diameter of 4. Similarity of the molecules was quantified by the Tanimoto-similarity measure. The range of applied similarity threshold t is 0.30 ≤ t ≤ 1.00 and t was incremented in steps of 0.01. (a) The number of edges in the similarity network in the function of the similarity threshold. (b) The average clustering coefficient (ACC) in the function of the applied similarity threshold. The obvious local maximum of the ACC vs. threshold curve is at threshold t α = 0.48. The value of the associated ACC(t α) is 0.5979. [file 13321_2016_127_MOESM8_ESM.docx]
