# Supplementary material for: Impact of similarity threshold on the topology of molecular similarity networks and clustering outcomes
Source: J Cheminform. 2016 Mar 30;8:16. doi: 10.1186/s13321-016-0127-5 (PMC4812625; doi:10.1186/s13321-016-0127-5)

Additional file 9: Figure S9: Number of clusters and singletons in the function of the selected threshold, WOMBAT dataset. Fingerprint: ECFP_4, similarity measure: Tanimoto similarity-coefficient, clustering algorithm: InfoMap, similarity threshold *t* incremented in steps of 0.01 in the range of *0.30 ≤ t ≤ 1.00.* (a) Number of clusters excluding singletons. The highest number of clusters, 18120, is observed at *t = 0.72*. (b) Number of clusters including singletons. (c) Number of singletons.


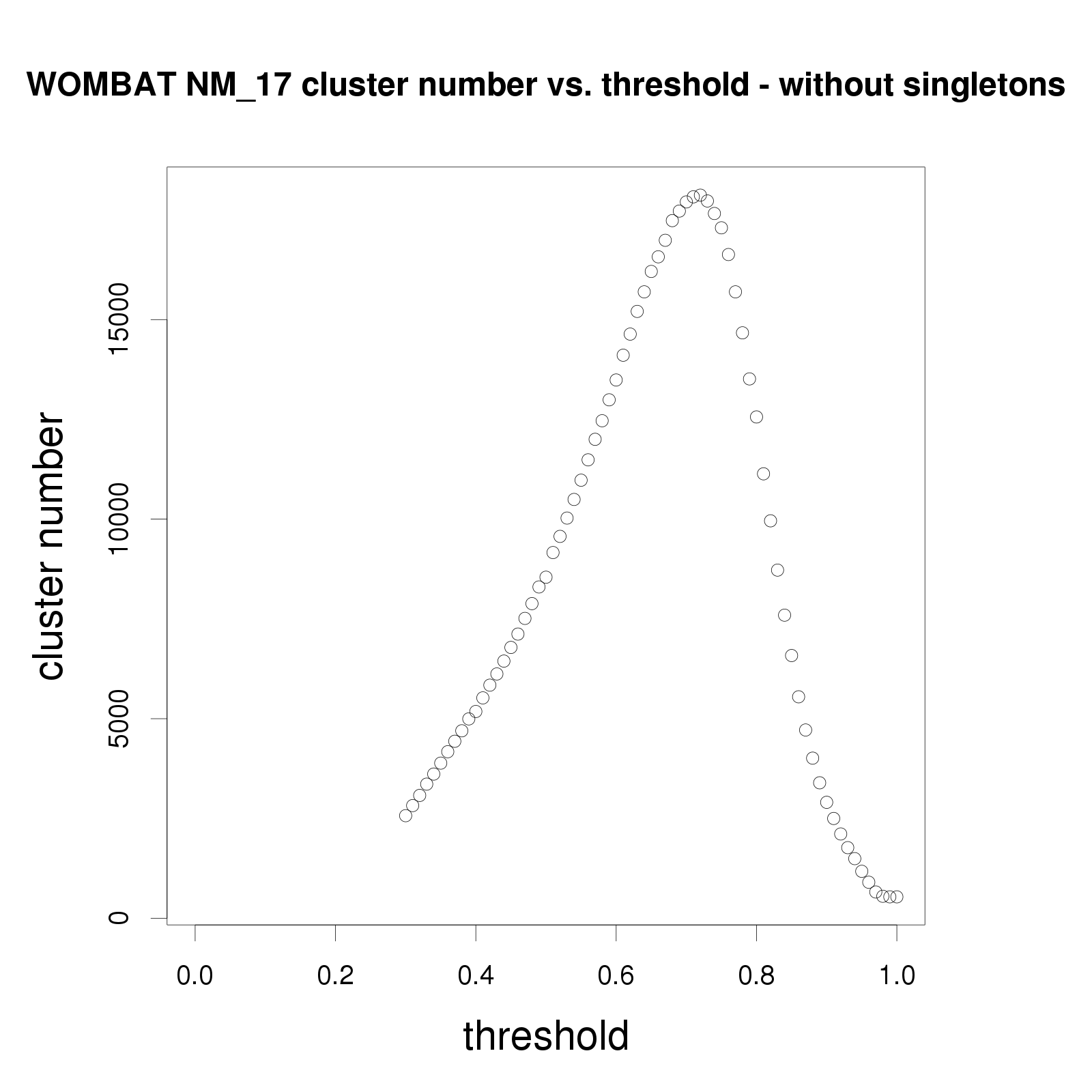


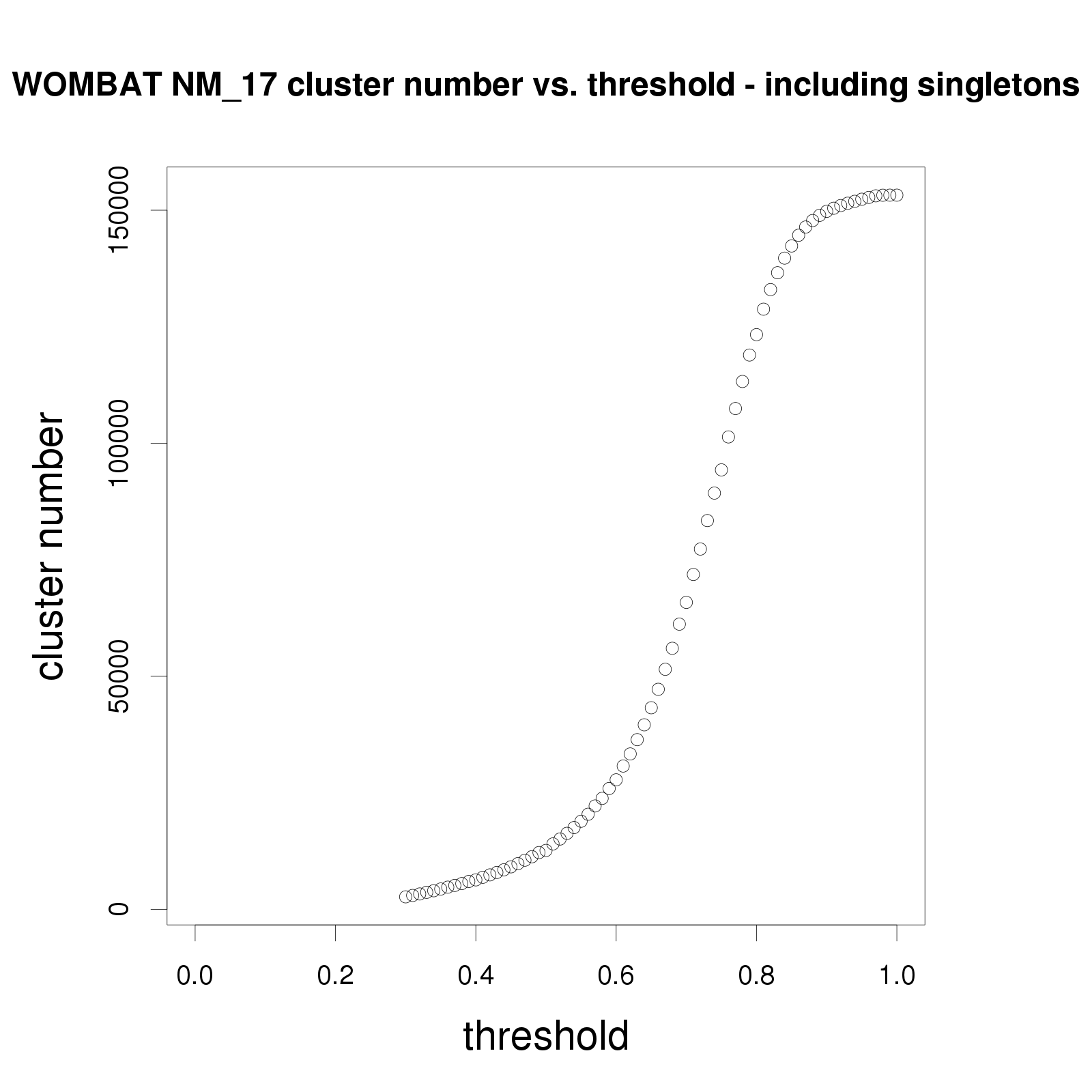


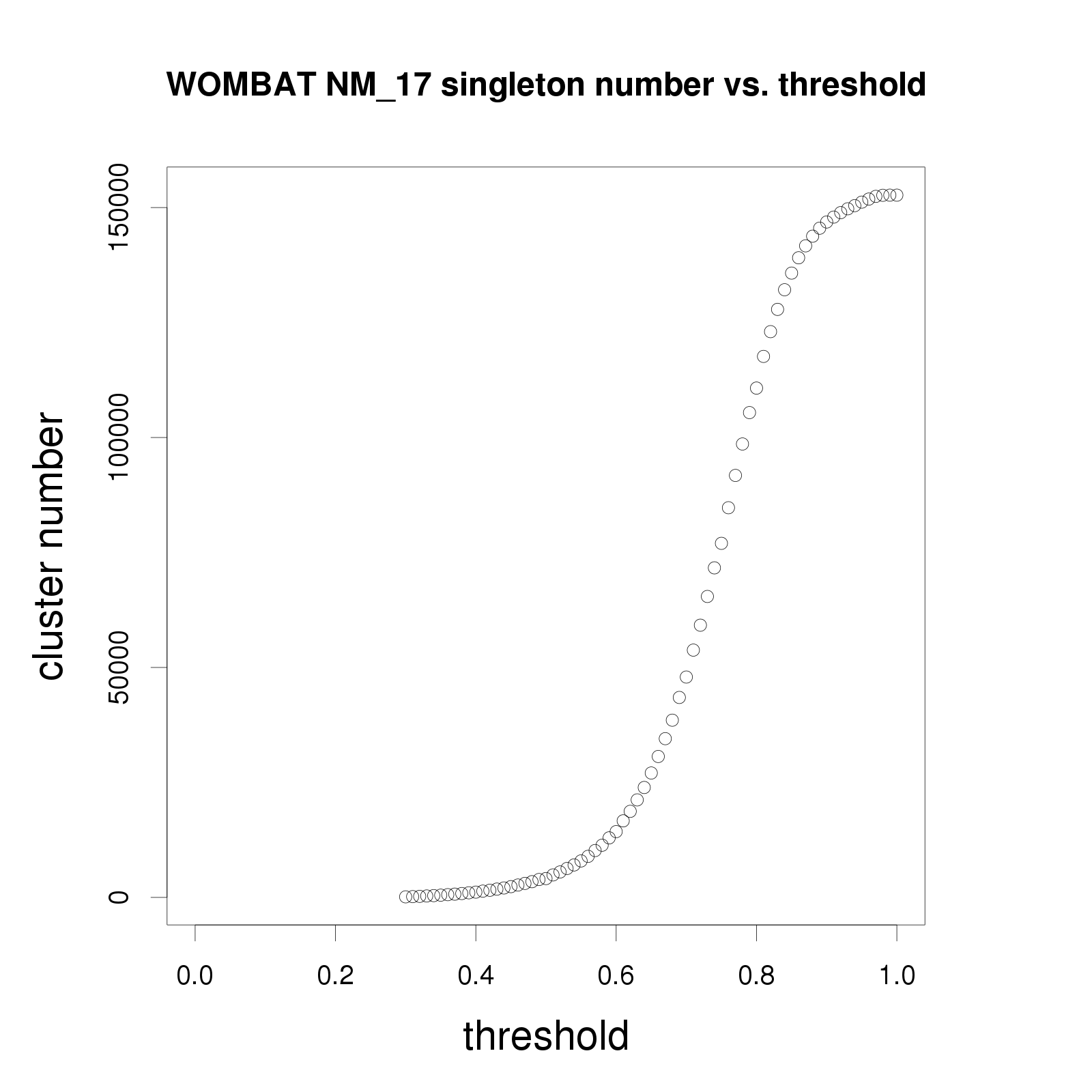

Supplement: Supplementary file 9 — 10.1186/s13321-016-0127-5 Number of clusters and singletons in the function of the selected threshold, WOMBAT dataset. Fingerprint: ECFP_4, similarity measure: Tanimoto similarity-coefficient, clustering algorithm: InfoMap, similarity threshold t incremented in steps of 0.01 in the range of 0.30 ≤ t ≤ 1.00. (a) Number of clusters excluding singletons. The highest number of clusters, 18,120, is observed at t = 0.72. (b) Number of clusters including singletons. (c) Number of singletons. [file 13321_2016_127_MOESM9_ESM.docx]
