# Supplementary material for: Impact of similarity threshold on the topology of molecular similarity networks and clustering outcomes
Source: J Cheminform. 2016 Mar 30;8:16. doi: 10.1186/s13321-016-0127-5 (PMC4812625; doi:10.1186/s13321-016-0127-5)

Additional file 11: Figure S11: Number of clusters and singletons in the function of the selected threshold, SCL dataset. Fingerprint: ECFP_4, similarity measure: Tanimoto similarity-coefficient, clustering algorithm: InfoMap, similarity threshold *t* incremented in steps of 0.01 in the range of *0.00 ≤ t ≤ 0.91.* Note, that above *t = 0.91* the similarity network only consists of singletons, therefore the respective experimental points are not displayed on the graph. (a) Number of clusters excluding singletons. (b) Number of clusters including singletons. (c) Number of singletons.


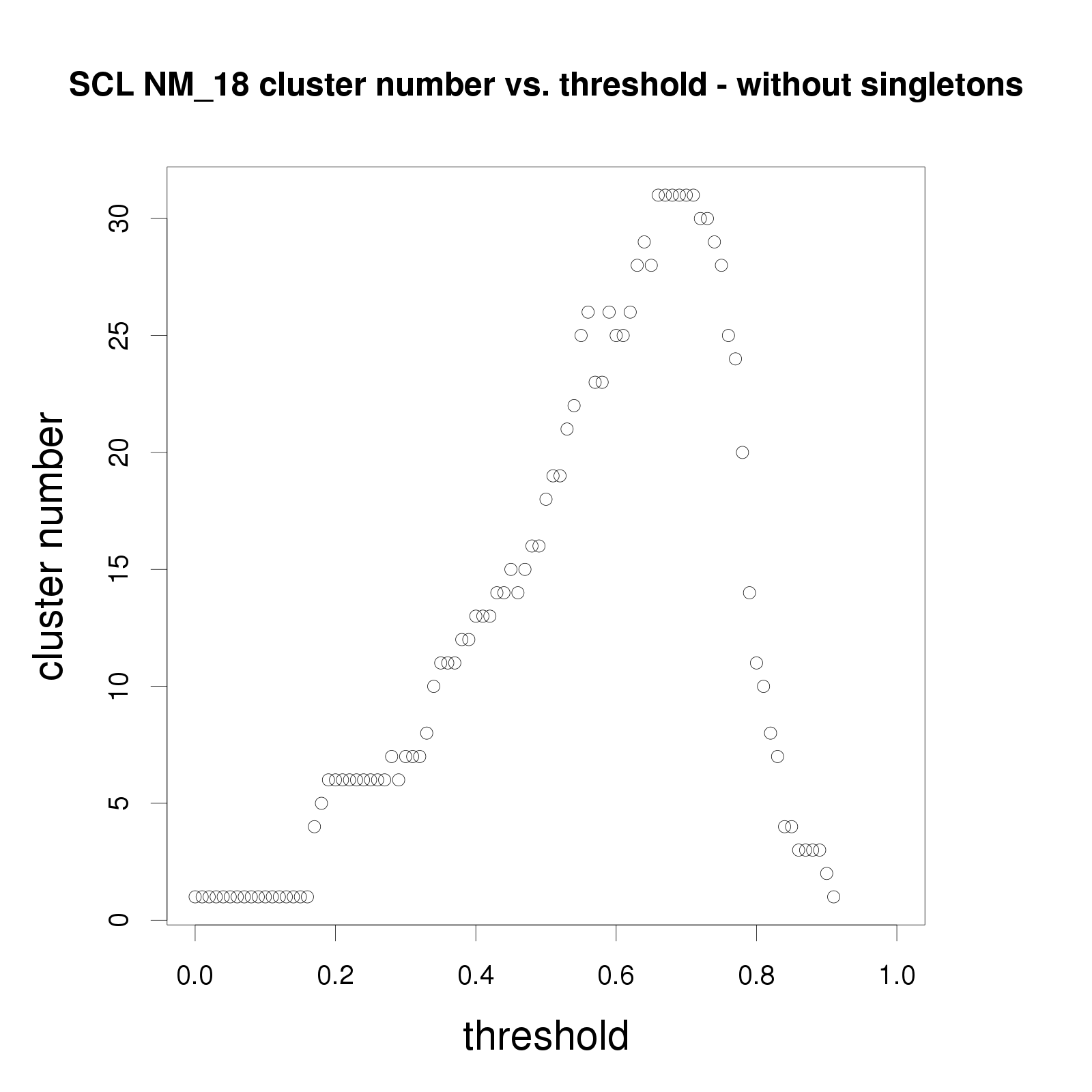


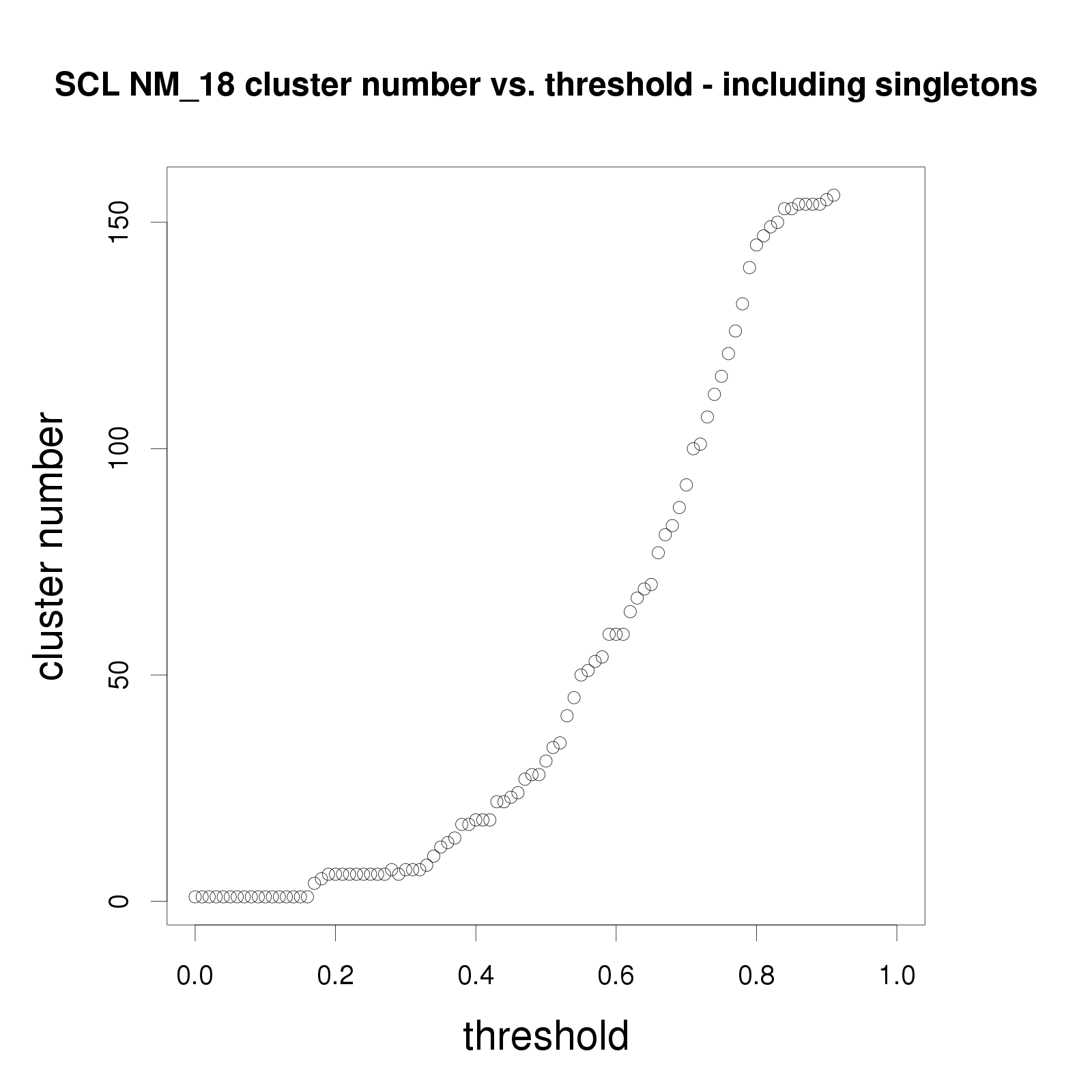


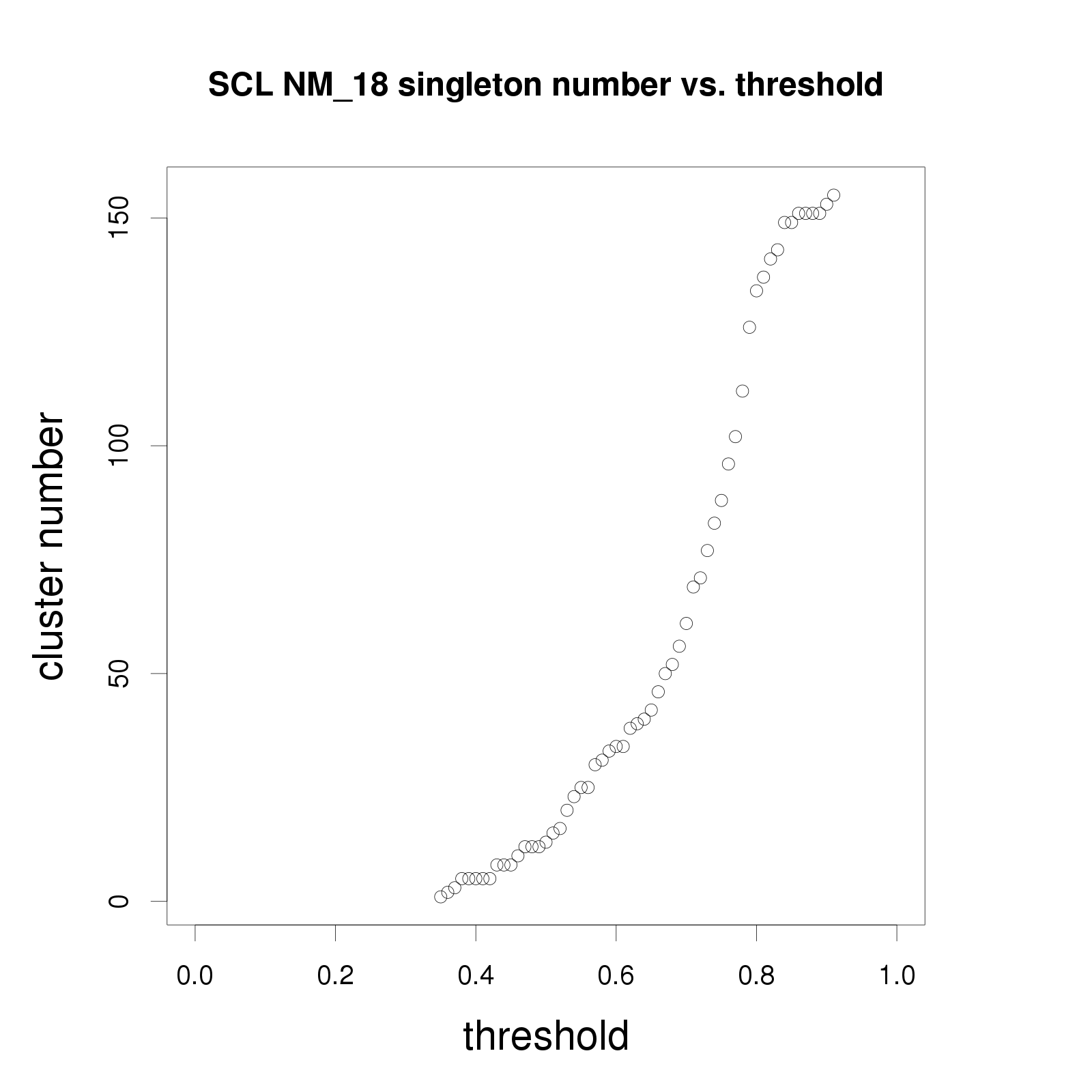

Supplement: Supplementary file 11 — 10.1186/s13321-016-0127-5 Number of clusters and singletons in the function of the selected threshold, SCL dataset. Fingerprint: ECFP_4, similarity measure: Tanimoto similarity-coefficient, clustering algorithm: InfoMap, similarity threshold t incremented in steps of 0.01 in the range of 0.00 ≤ t ≤ 0.91. Note, that above t = 0.91 the similarity network only consists of singletons, therefore the respective experimental points are not displayed on the graph. (a) Number of clusters excluding singletons. (b) Number of clusters including singletons. (c) Number of singletons. [file 13321_2016_127_MOESM11_ESM.docx]
