# Supplementary material for: Impact of similarity threshold on the topology of molecular similarity networks and clustering outcomes
Source: J Cheminform. 2016 Mar 30;8:16. doi: 10.1186/s13321-016-0127-5 (PMC4812625; doi:10.1186/s13321-016-0127-5)
Supplement: Supplementary file 12 — 10.1186/s13321-016-0127-5 Illustrative cluster of WOMBAT dataset at threshold = 0.40. File name: wombat_nm17_cid_1178_t_alpha_0.40_pub.pdf . Shown are the molecules of cluster 1178 of WOMBAT dataset produced at the obvious local maximum of the ACC vs. threshold curve at threshold t α = 0.40. PDF generated by ChemAxon’s mview utility. [file 13321_2016_127_MOESM12_ESM.pdf]

1

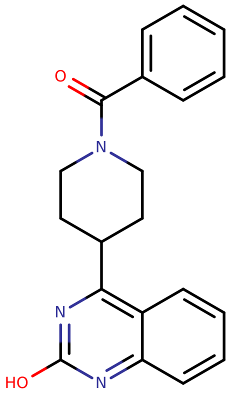

278782

2

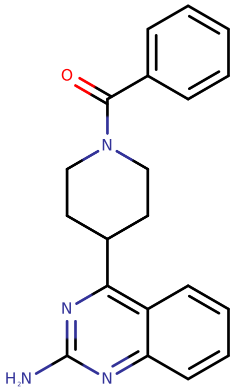

278783

3

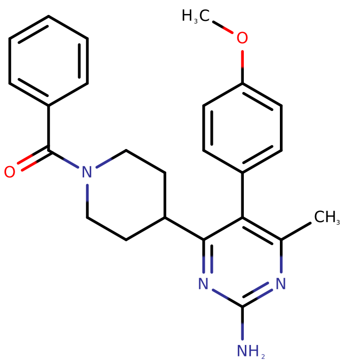

278803

4

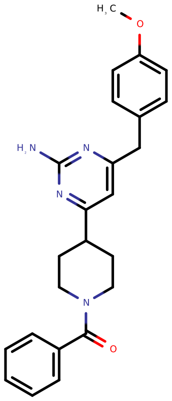

278802

5

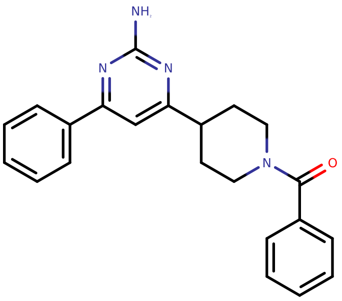

278801

6

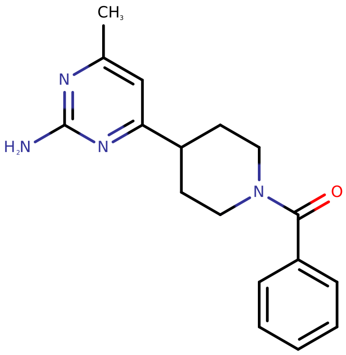

278800

7

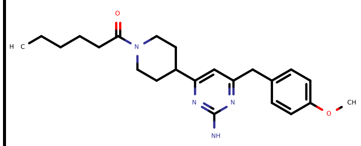

278807

8

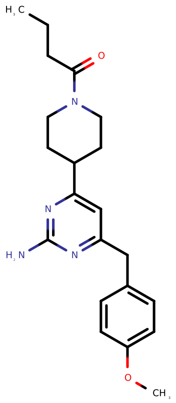

278806

9

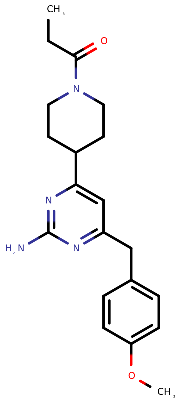

278805

10

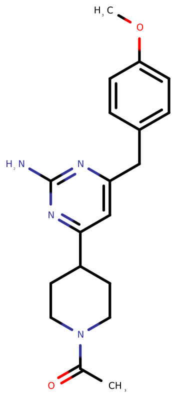

278804

11

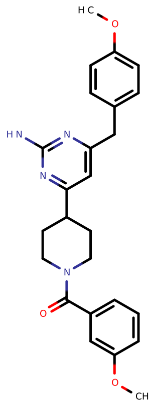

278811

12

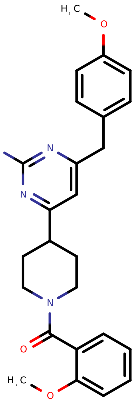

278810

13

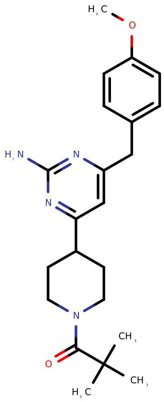

278809

14

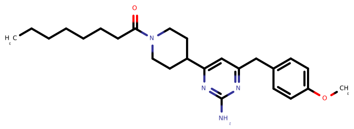

278808

15

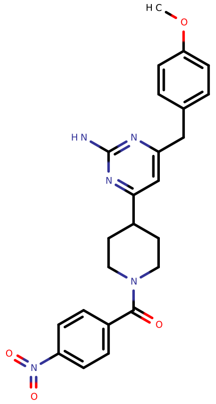

278815

16

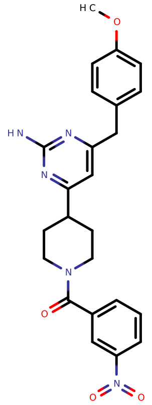

278814

17	18	19	20
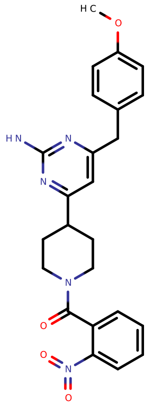	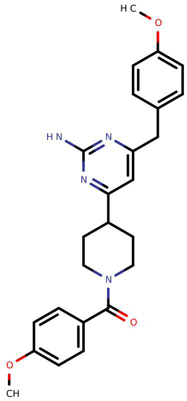	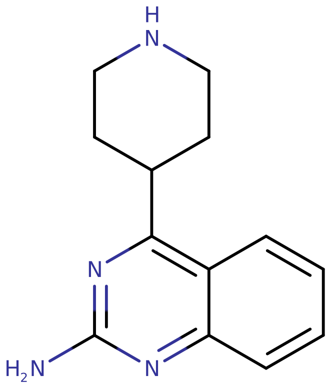	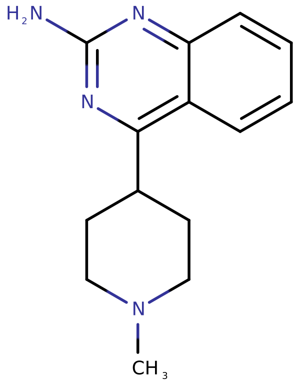
278813	278812	278786	278787
**21**	**22**	**23**	**24**
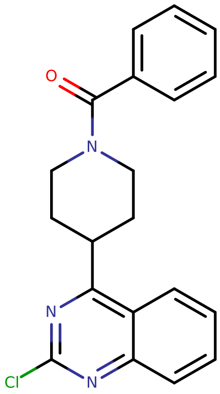	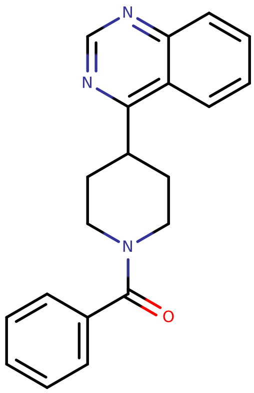	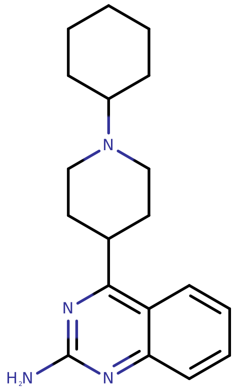	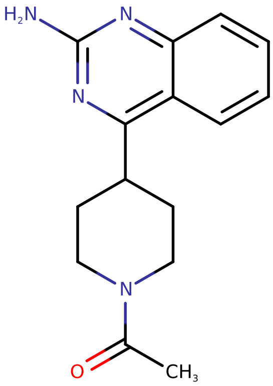
278784	278785	278790	278791
**25**	**26**	**27**	**28**
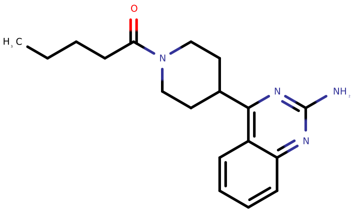	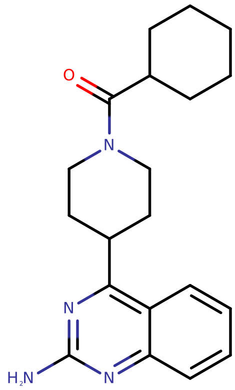	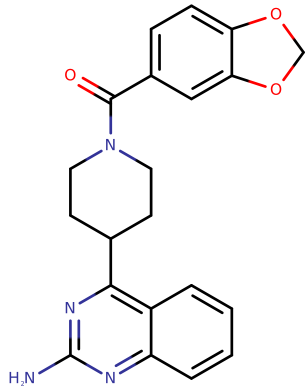	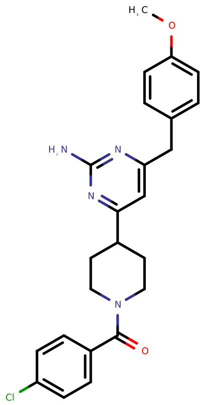
278792	278793	278796	278816
**29**	**30**	**31**	**32**
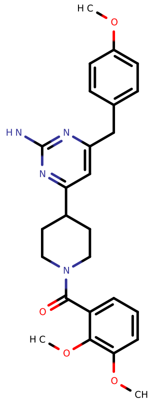	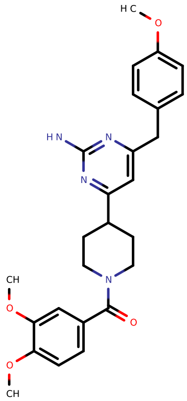	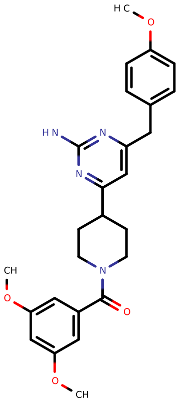	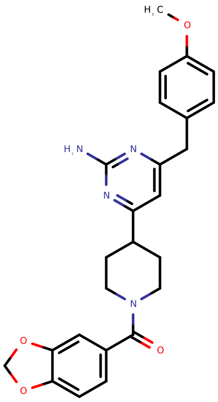
278817	278818	278819	278820

33	34	35	36
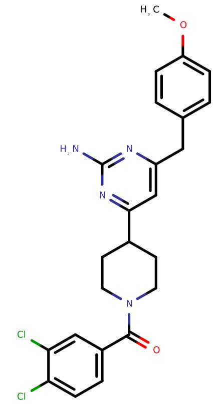	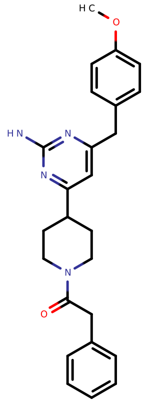	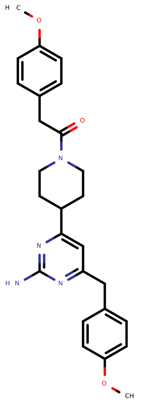	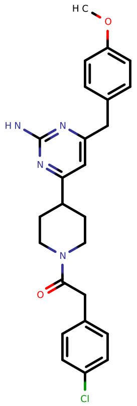
278821	278822	278823	278824
**37**	**38**	**39**	
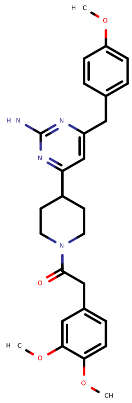	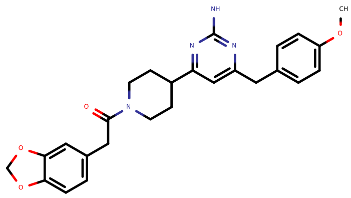	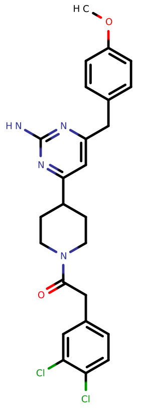	
278825	278826	278827	
