# Supplementary material for: Impact of similarity threshold on the topology of molecular similarity networks and clustering outcomes
Source: J Cheminform. 2016 Mar 30;8:16. doi: 10.1186/s13321-016-0127-5 (PMC4812625; doi:10.1186/s13321-016-0127-5)
Supplement: Supplementary file 13 — 10.1186/s13321-016-0127-5 Illustrative cluster of WOMBAT dataset at threshold = 0.72. File name: wombat_nm17_cid_505_t_0.72_pub.pdf . Shown are the molecules of cluster 505 of WOMBAT dataset produced at threshold t = 0.72 associated with the highest number of clusters (singletons excluded). PDF generated by ChemAxon’s mview utility. [file 13321_2016_127_MOESM13_ESM.pdf]

1

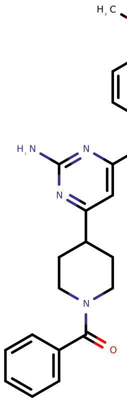

278802

2

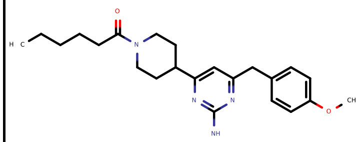

278807

3

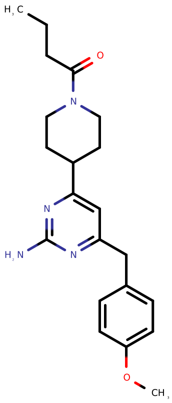

278806

4

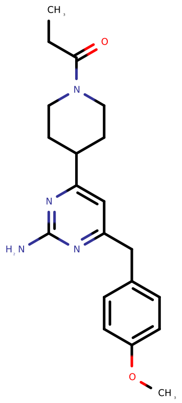

278805

5

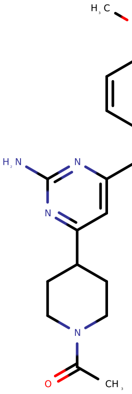

278804

6

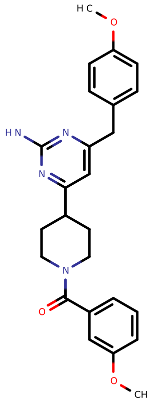

278811

7

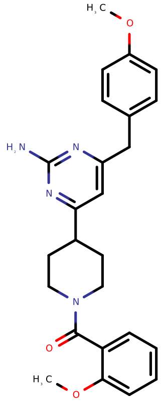

278810

8

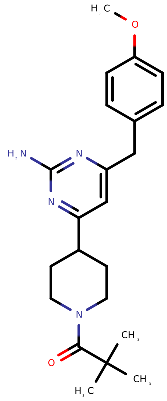

278809

9

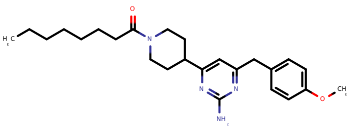

278808

10

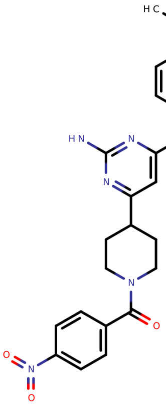

278815

11

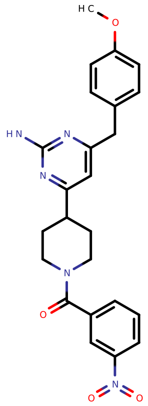

278814

12

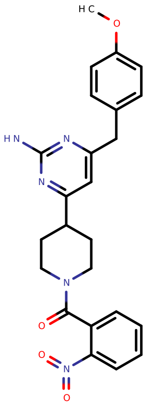

278813

13

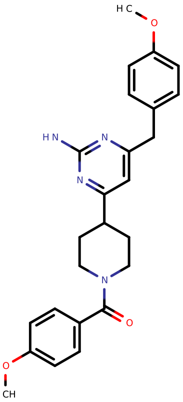

278812

14

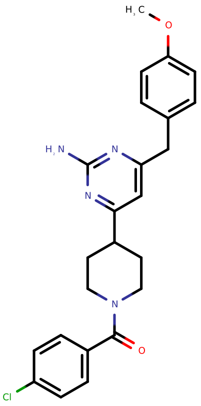

278816

15

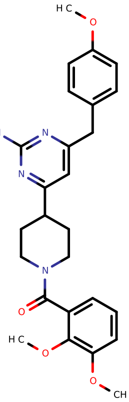

278817

16

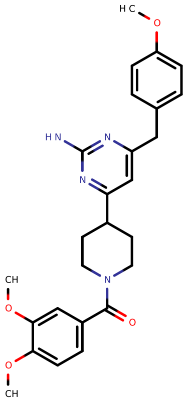

278818

17

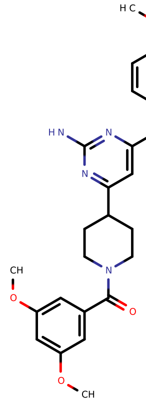

278819

18

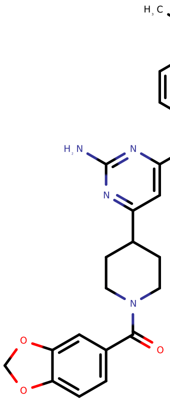

278820

19

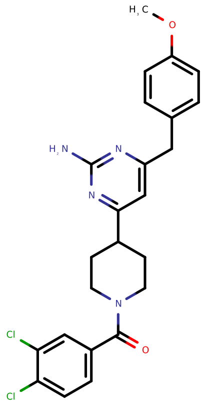

278821

20

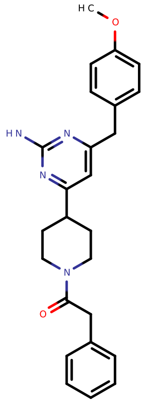

278822

21

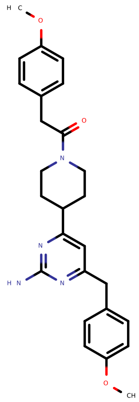

278823

22

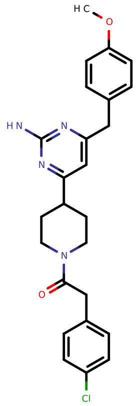

278824

23

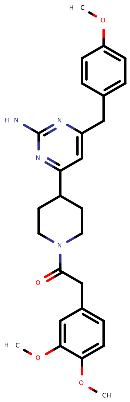

278825

24

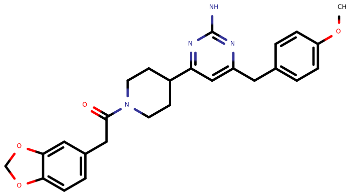

278826

25

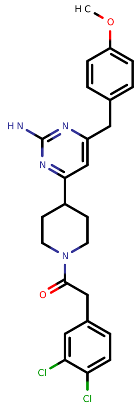

278827
