# Supplementary material for: Impact of similarity threshold on the topology of molecular similarity networks and clustering outcomes
Source: J Cheminform. 2016 Mar 30;8:16. doi: 10.1186/s13321-016-0127-5 (PMC4812625; doi:10.1186/s13321-016-0127-5)
Supplement: Supplementary file 14 — 10.1186/s13321-016-0127-5 Illustrative cluster of PubChem MLSMR dataset at the threshold = 0.50. File name: mlsmr_nm16_t_alpha_0.50_cid_674_pub.pdf. Shown are the molecules of cluster 674 of PubChem MLSMR dataset produced at the obvious local maximum of the ACC vs. threshold curve at threshold t α = 0.50. PDF generated by ChemAxon’s mview utility. [file 13321_2016_127_MOESM14_ESM.pdf]

1	2	3	4
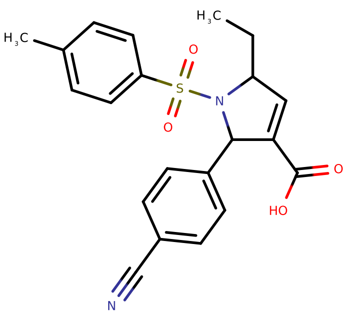	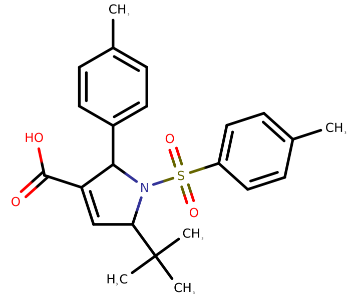	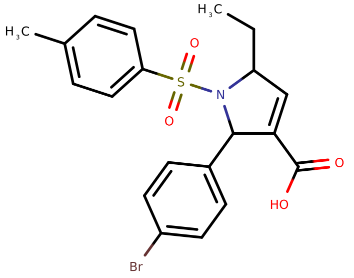	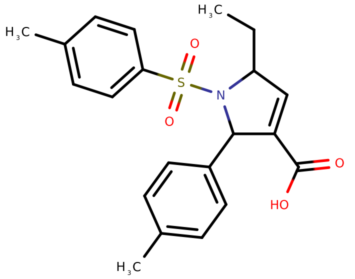
6610119	6610115	6610146	6610109
**5**	**6**	**7**	**8**
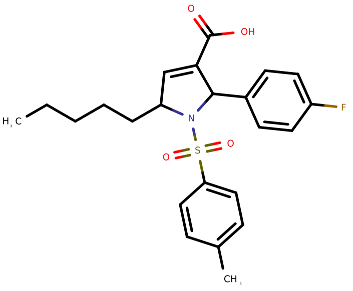	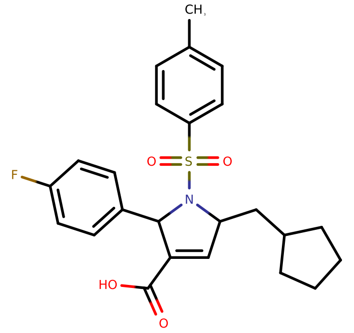	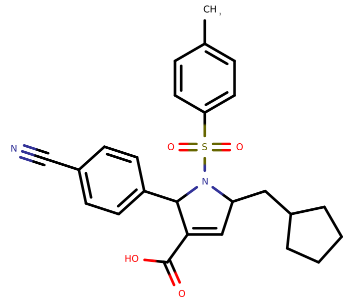	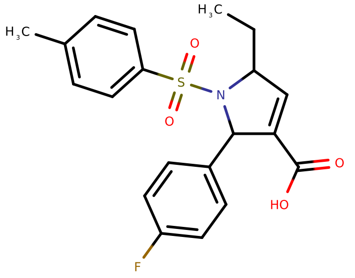
44263586	44263587	44263584	44263585
**9**	**10**	**11**	**12**
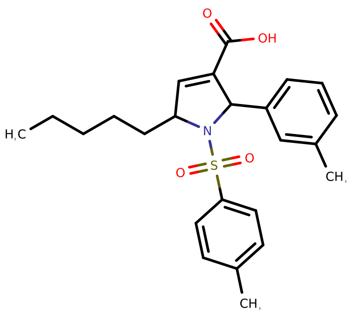	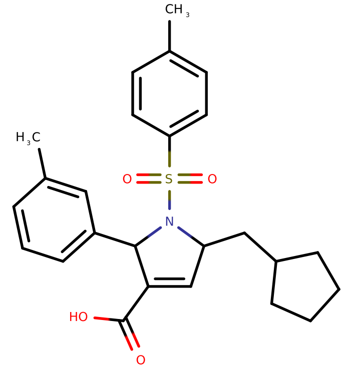	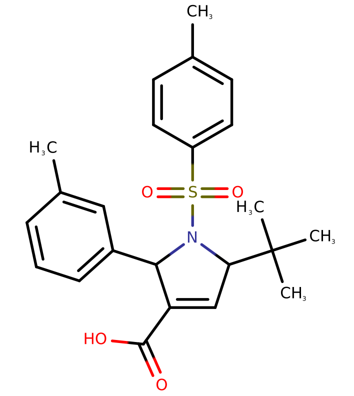	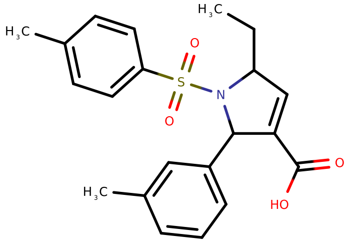
44263590	44263591	44263588	44263589
**13**	**14**	**15**	**16**
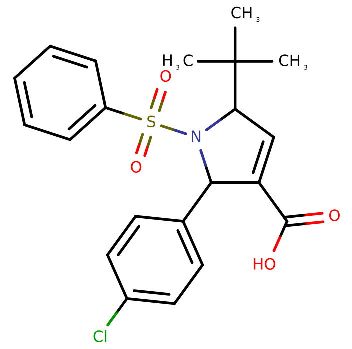	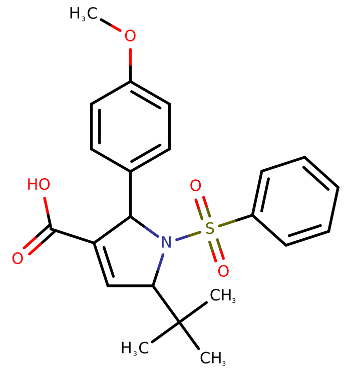	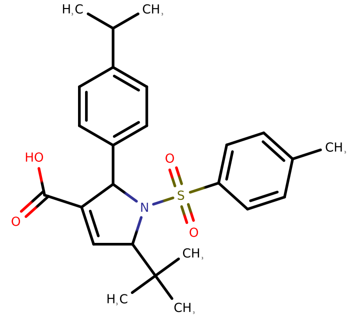	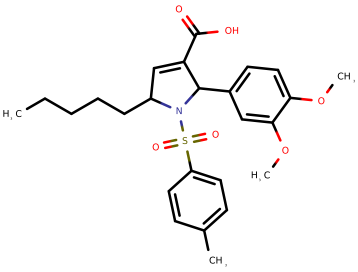
44263594	44263595	44263593	44263569

17

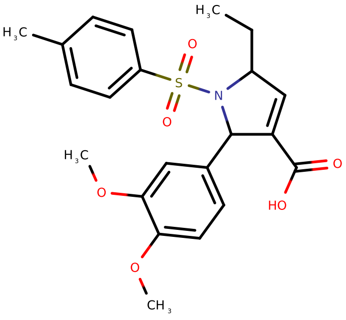

44263568

18

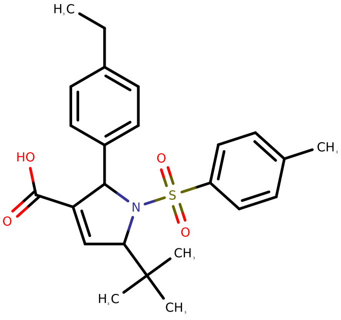

44263571

19

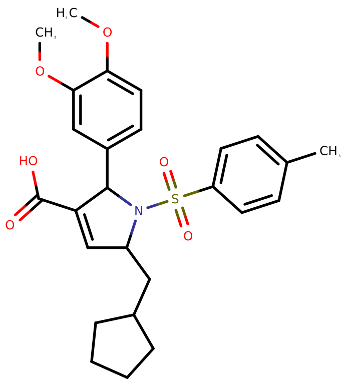

44263570

20

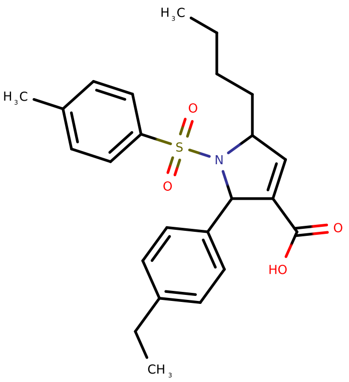

44263573

21

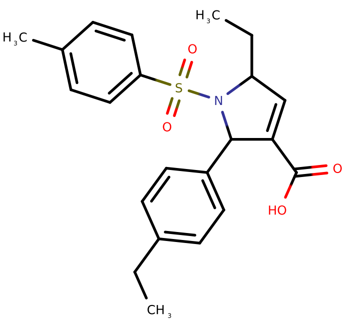

44263572

22

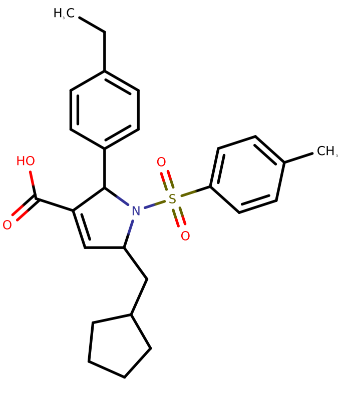

44263575

23

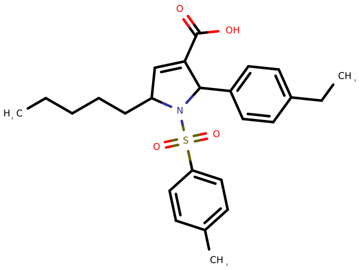

44263574

24

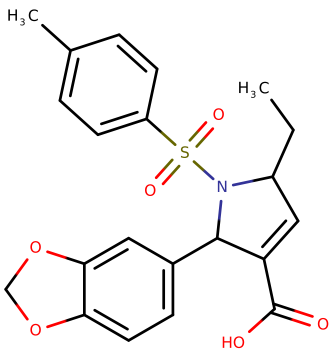

44263577

25

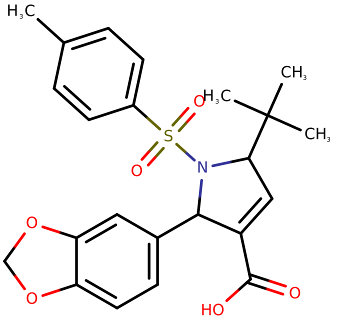

44263576

26

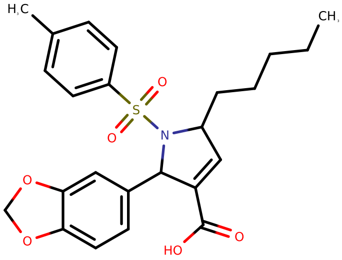

44263579

27

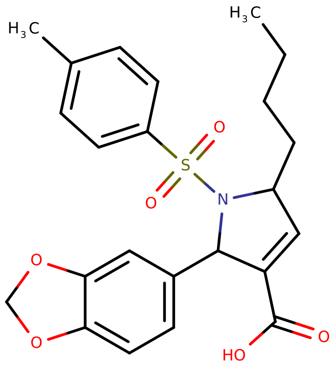

44263578

28

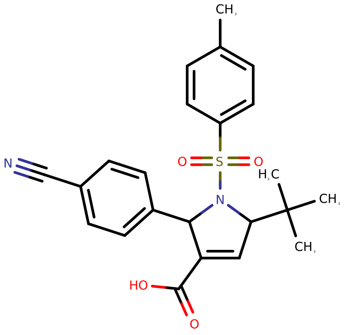

44263581

29

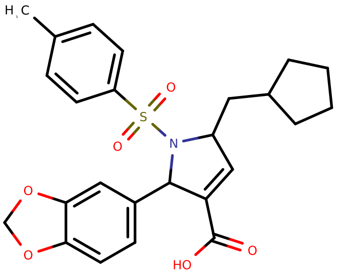

44263580

30

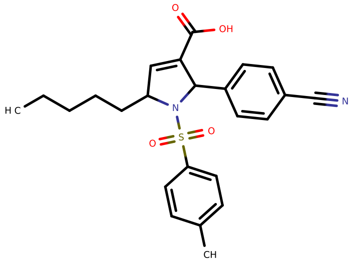

44263583

31

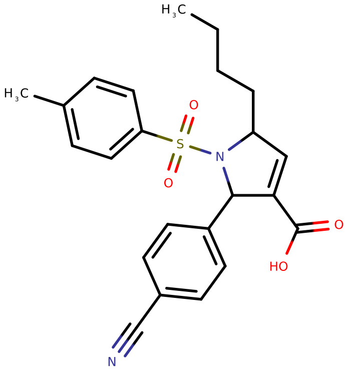

44263582

32

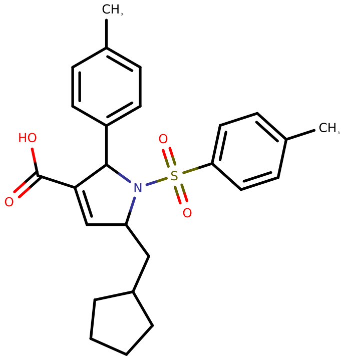

44263552

33

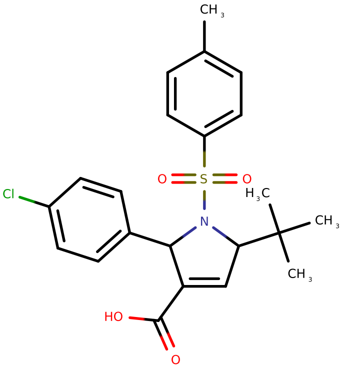

44263553

34

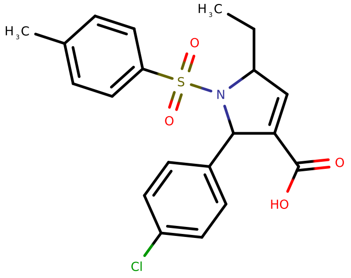

44263554

35

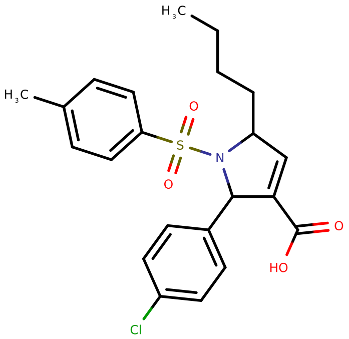

44263555

36

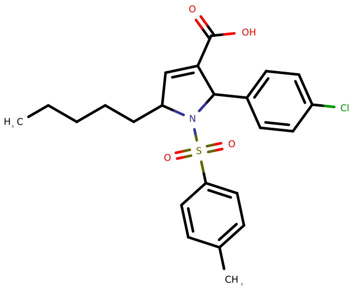

44263556

37

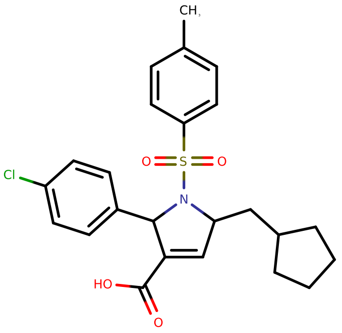

44263557

38

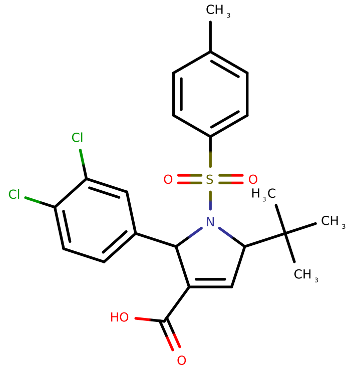

44263558

39

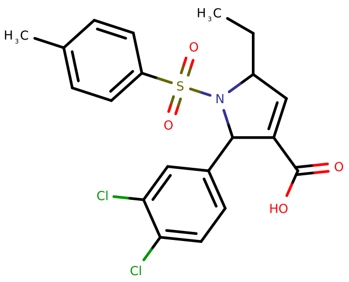

44263559

40

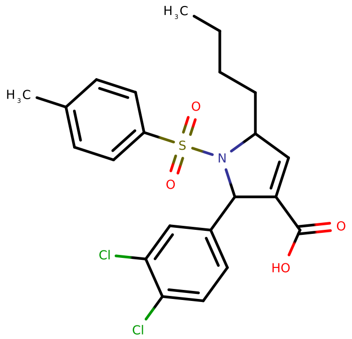

44263560

41

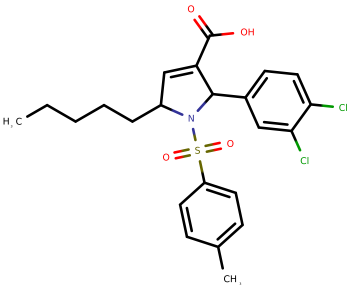

44263561

42

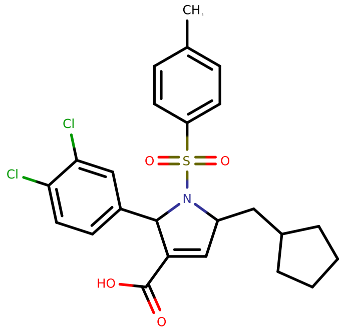

44263562

43

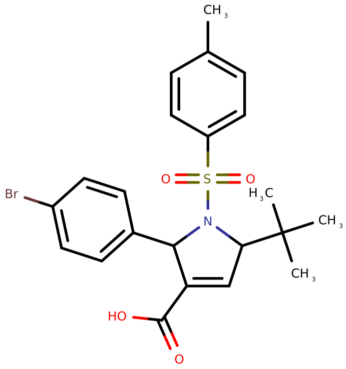

44263563

44

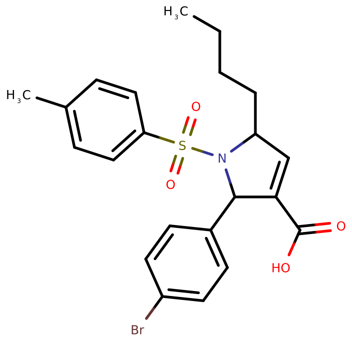

44263564

45

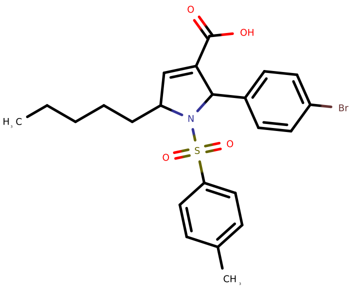

44263565

46

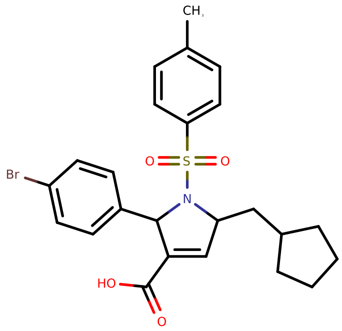

44263566

47

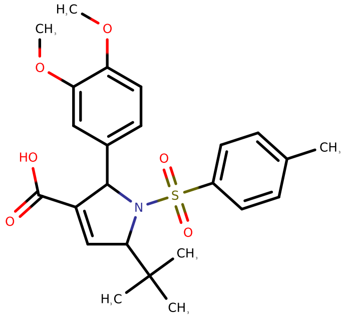

44263567

48

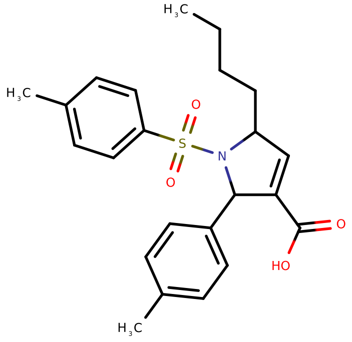

44263550

49			
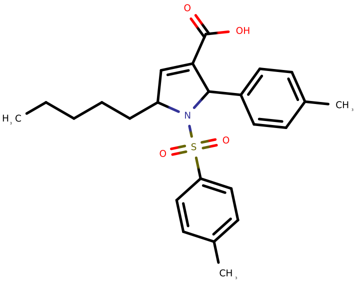			
44263551			
