# Supplementary material for: Impact of similarity threshold on the topology of molecular similarity networks and clustering outcomes
Source: J Cheminform. 2016 Mar 30;8:16. doi: 10.1186/s13321-016-0127-5 (PMC4812625; doi:10.1186/s13321-016-0127-5)
Supplement: Supplementary file 15 — 10.1186/s13321-016-0127-5 Illustrative cluster of PubChem MLSMR dataset at the threshold = 0.68. File name: mlsmr_nm16_t_0.68_cid_100_pub.pdf . Shown are the molecules of cluster 100 of PubChem MLSMR dataset produced at threshold t = 0.68 associated with the highest number of clusters (singletons excluded). PDF generated by ChemAxon’s mview utility. [file 13321_2016_127_MOESM15_ESM.pdf]

1	2	3	4
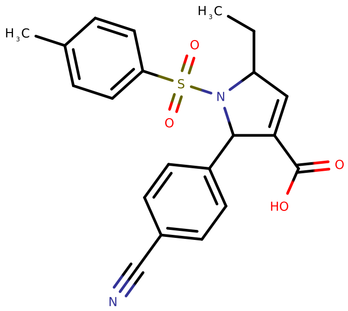	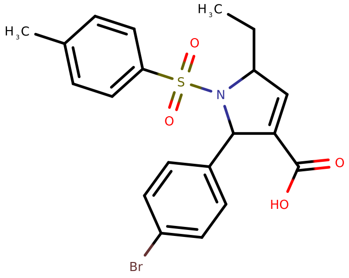	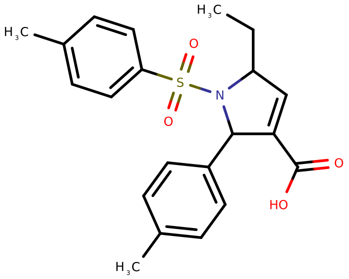	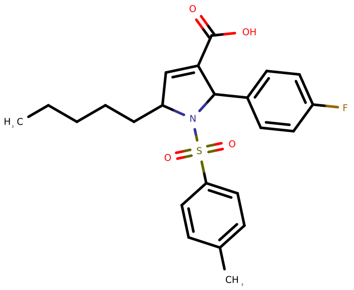
6610119	6610146	6610109	44263586
**5**	**6**	**7**	**8**
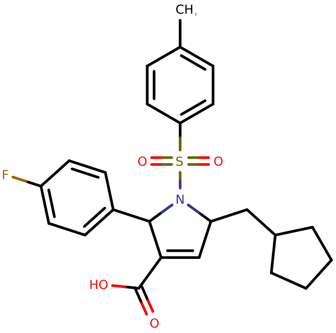	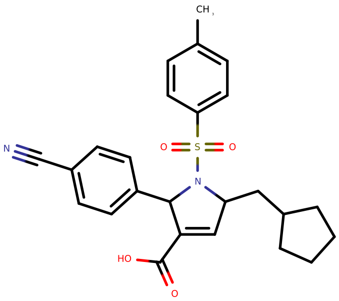	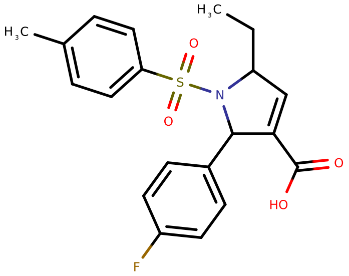	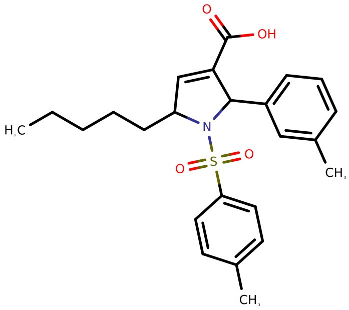
44263587	44263584	44263585	44263590
**9**	**10**	**11**	**12**
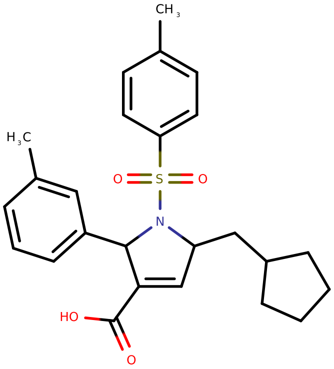	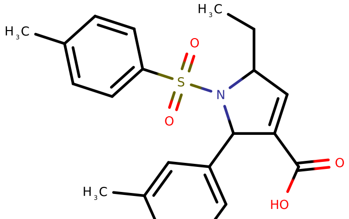	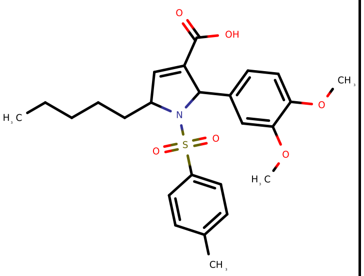	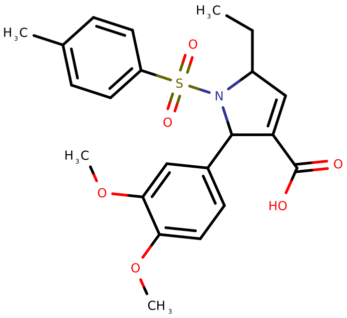
44263591	44263589	44263569	44263568
**13**	**14**	**15**	**16**
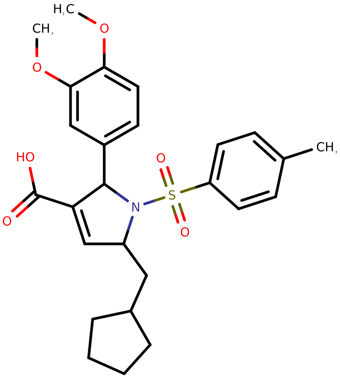	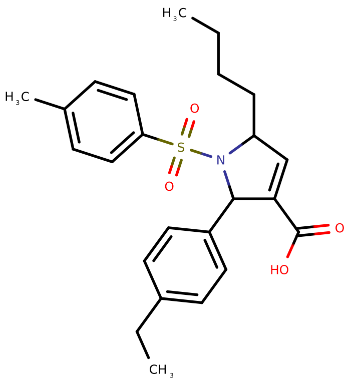	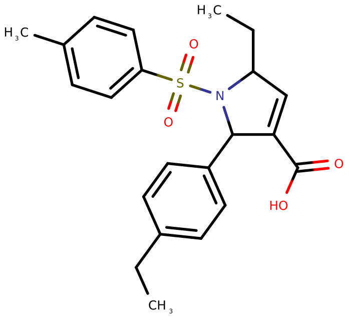	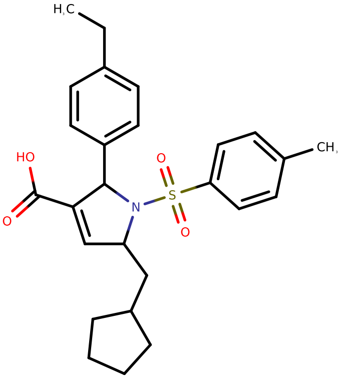
44263570	44263573	44263572	44263575

17	18	19	20
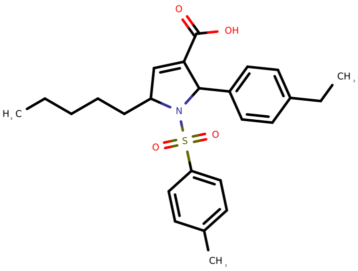	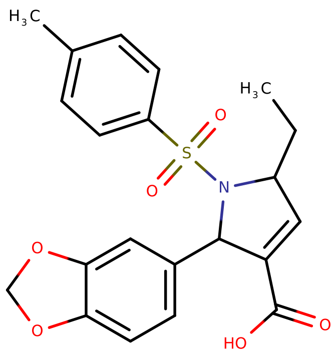	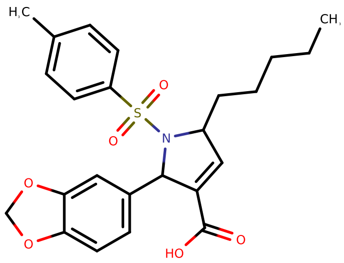	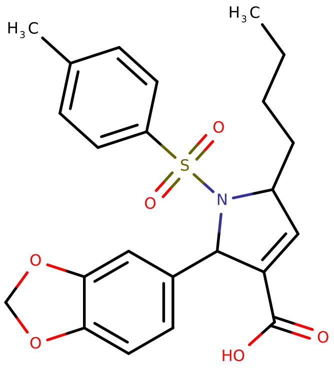
44263574	44263577	44263579	44263578
**21**	**22**	**23**	**24**
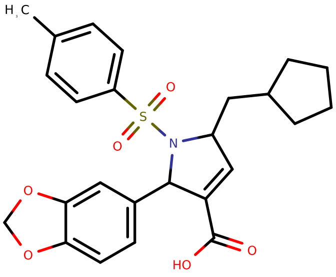	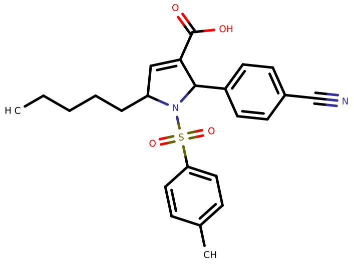	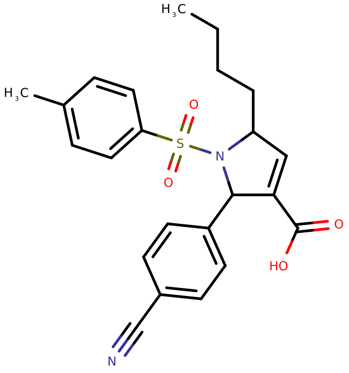	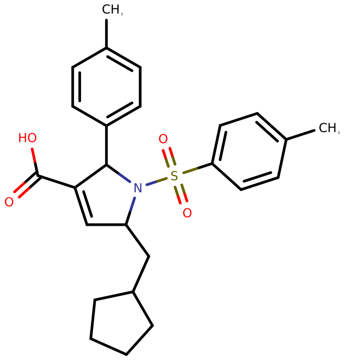
44263580	44263583	44263582	44263552
**25**	**26**	**27**	**28**
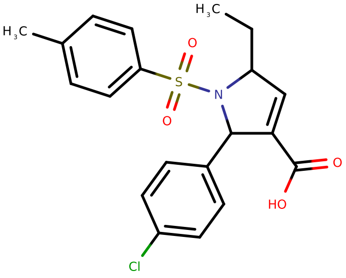	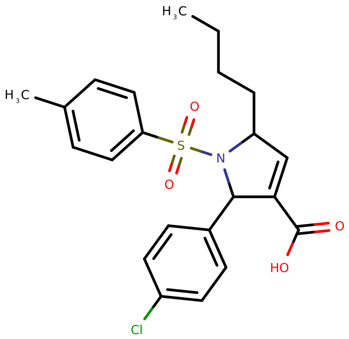	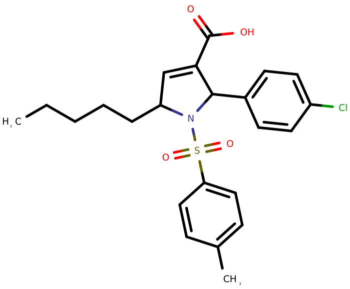	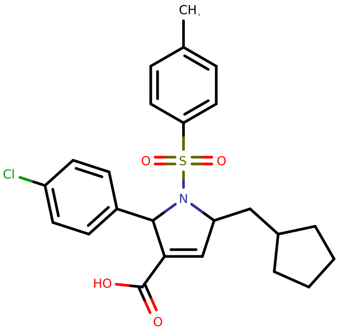
44263554	44263555	44263556	44263557
**29**	**30**	**31**	**32**
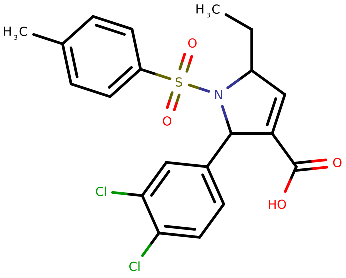	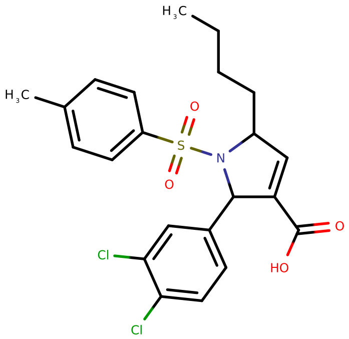	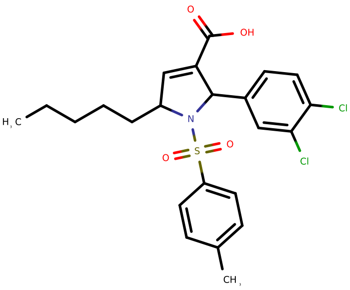	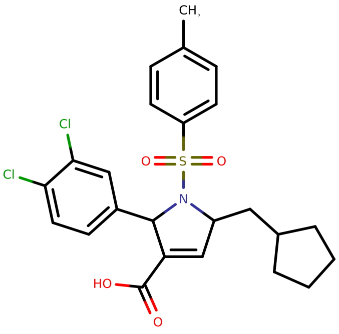
44263559	44263560	44263561	44263562

33	34	35	36	37
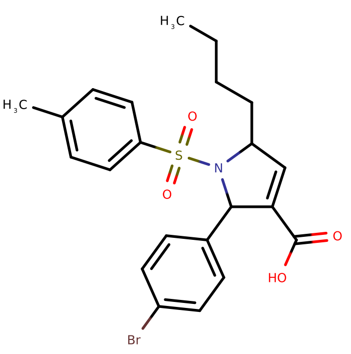	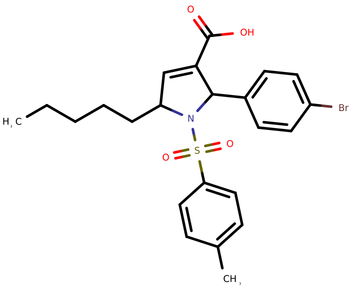	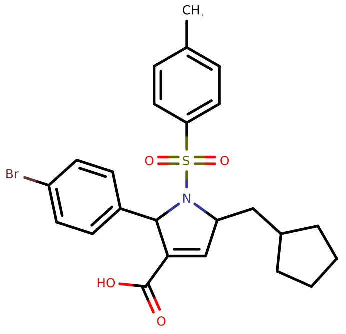	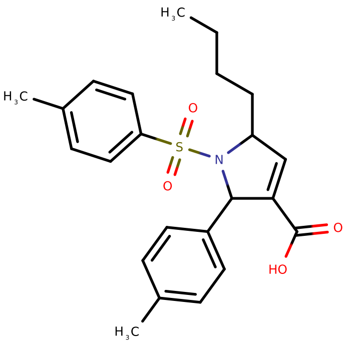	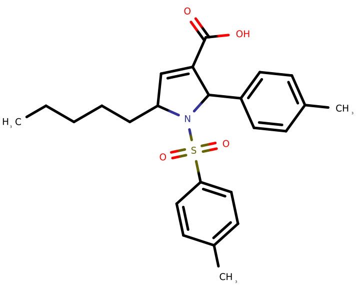
44263564	44263565	44263566	44263550	44263551
