# Supplementary material for: Impact of similarity threshold on the topology of molecular similarity networks and clustering outcomes
Source: J Cheminform. 2016 Mar 30;8:16. doi: 10.1186/s13321-016-0127-5 (PMC4812625; doi:10.1186/s13321-016-0127-5)

Additional file 16: Figure S12: The effect of the applied fingerprint on the network topology in the case of SCL dataset. Tanimoto similarity threshold was incremented by steps of 0.01 in the range of 0 to 1. The choice of molecular fingerpint generating method has a profound effect on both the *ACC(t)* and *edge number(t)* functions.


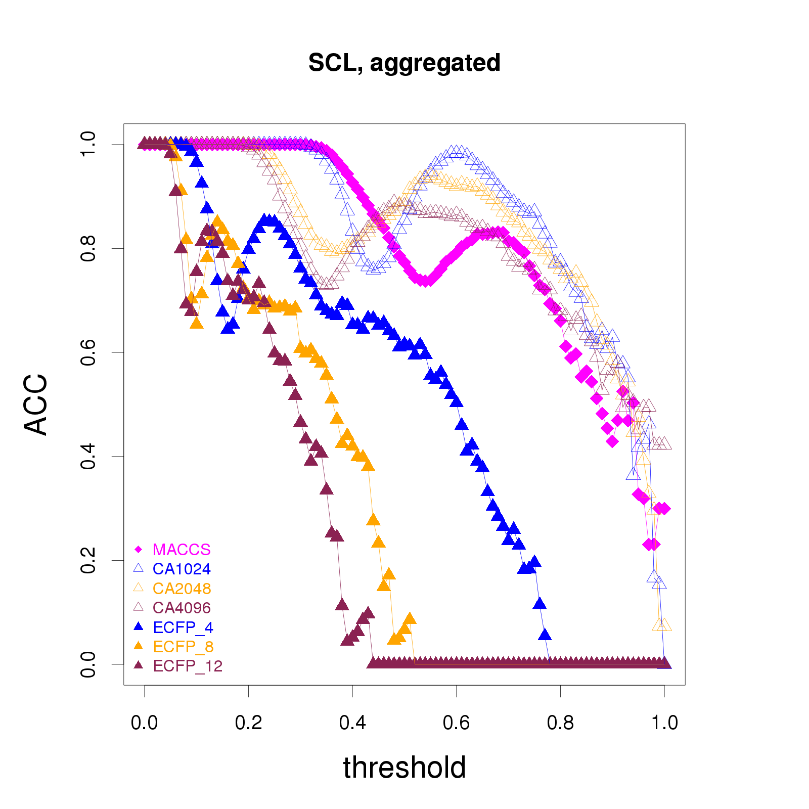


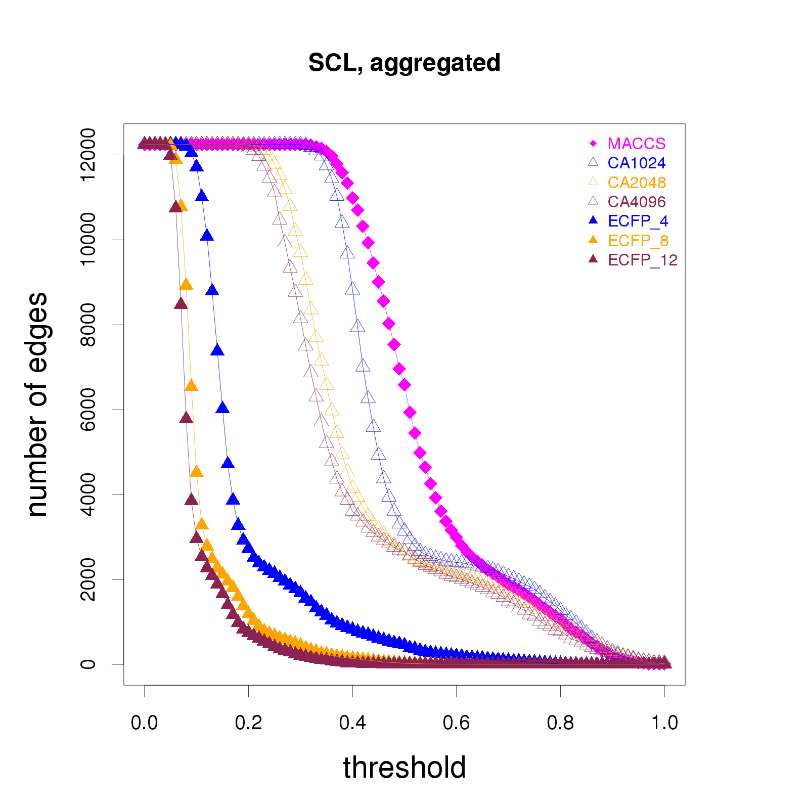

Supplement: Supplementary file 16 — 10.1186/s13321-016-0127-5 The effect of the applied fingerprint on the network topology in the case of SCL dataset. Tanimoto similarity threshold was incremented by steps of 0.01 in the range of 0 to 1. The choice of molecular fingerpint generating method has a profound effect on both the ACC(t) and EN(t) functions. [file 13321_2016_127_MOESM16_ESM.docx]
