# Supplementary material for: Impact of similarity threshold on the topology of molecular similarity networks and clustering outcomes
Source: J Cheminform. 2016 Mar 30;8:16. doi: 10.1186/s13321-016-0127-5 (PMC4812625; doi:10.1186/s13321-016-0127-5)

#### Additional file 19: Figure S15: First and second order derivatives of the number of edges vs. threshold function in the case of the PubChem MLSMR dataset. The aforementioned function is denoted by *f(x)*, and it’s first and second order functions by *f’(x)* and *f’’(x)*, respectively. The derivatives were approximated by numerical differentiation. The vertical line at threshold *t_γ_* denotes the threshold associated with the observed best clustering performance. First order derivatives: (a) using the forward-difference, (b) using the backward difference, (c) using the central difference. (d) Second order derivative.


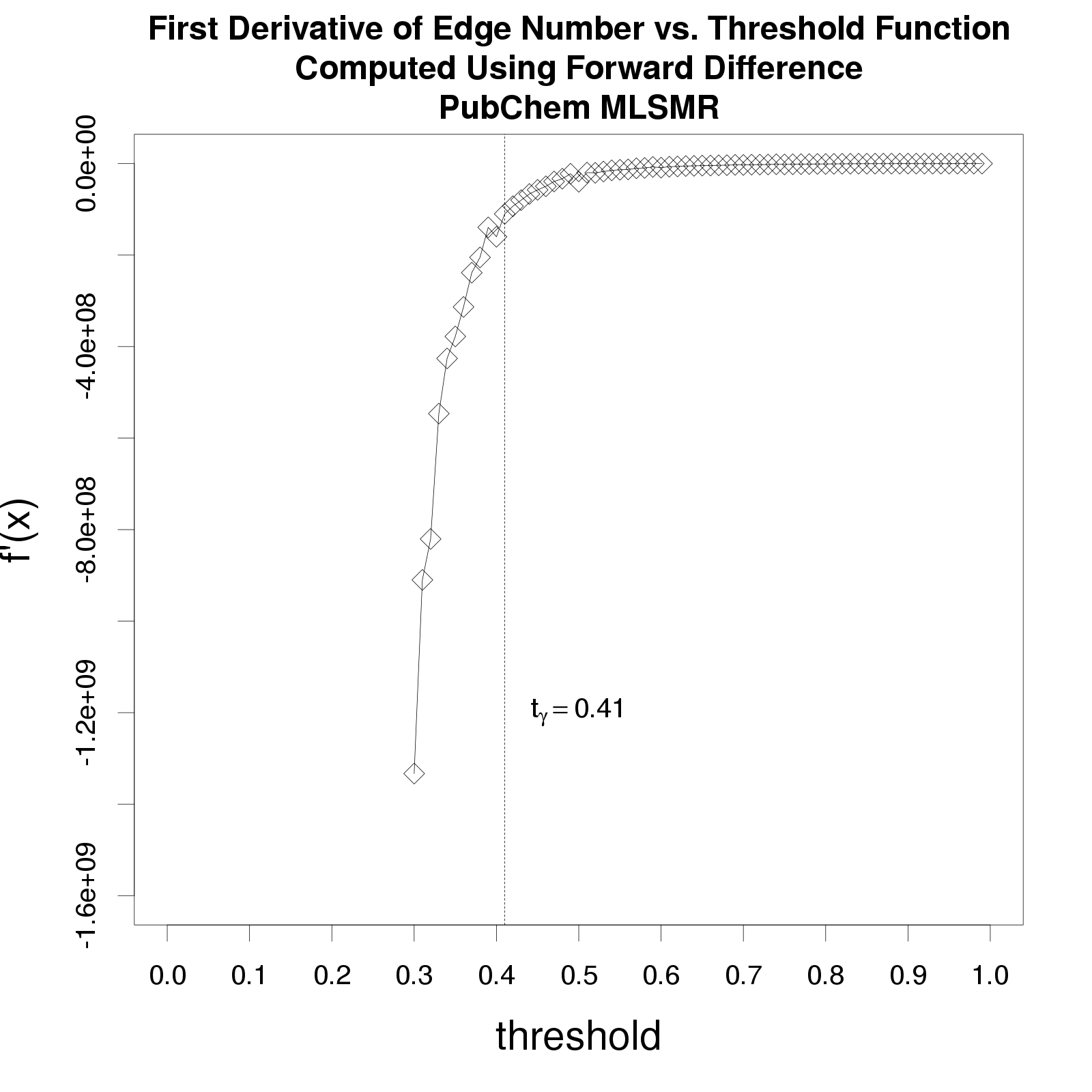


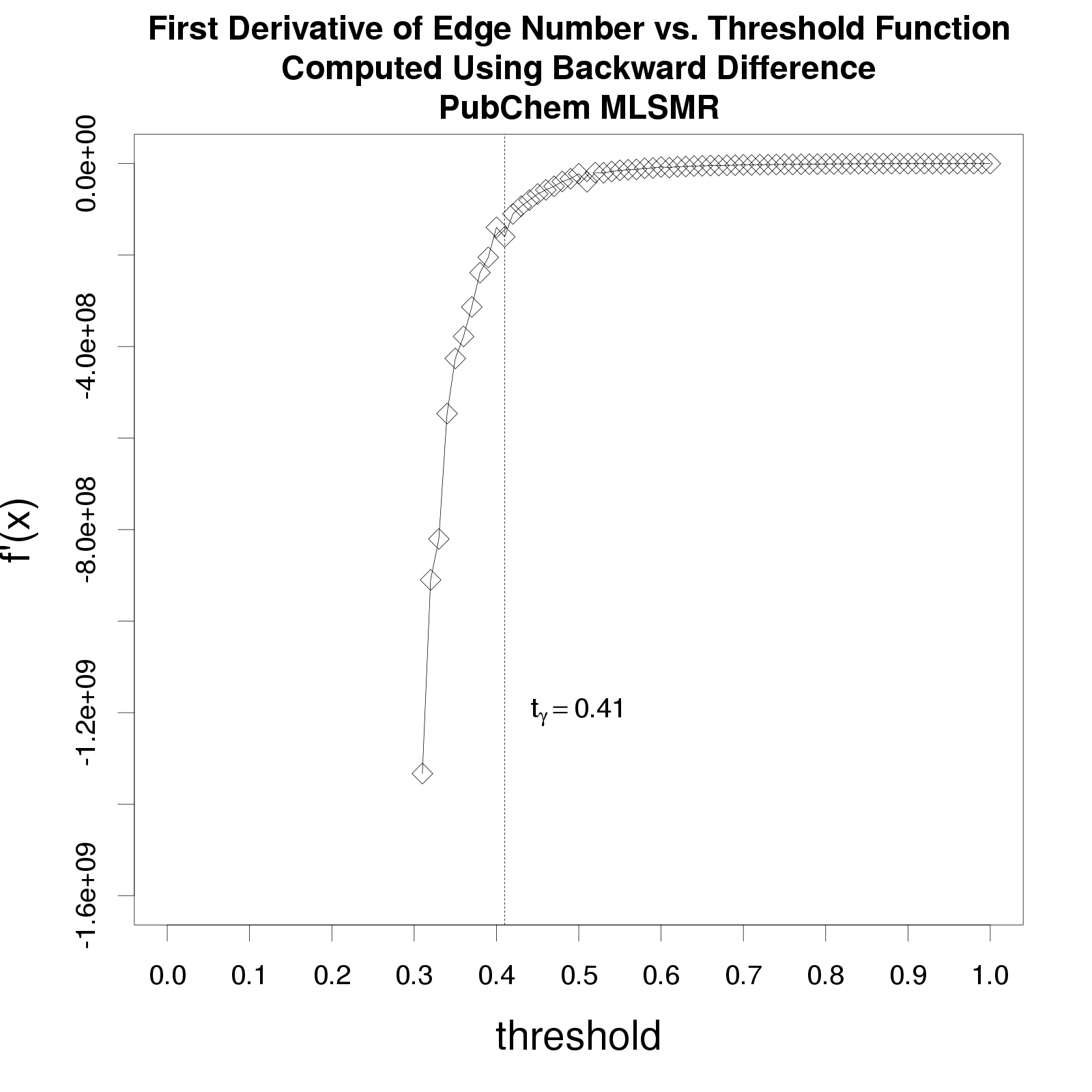


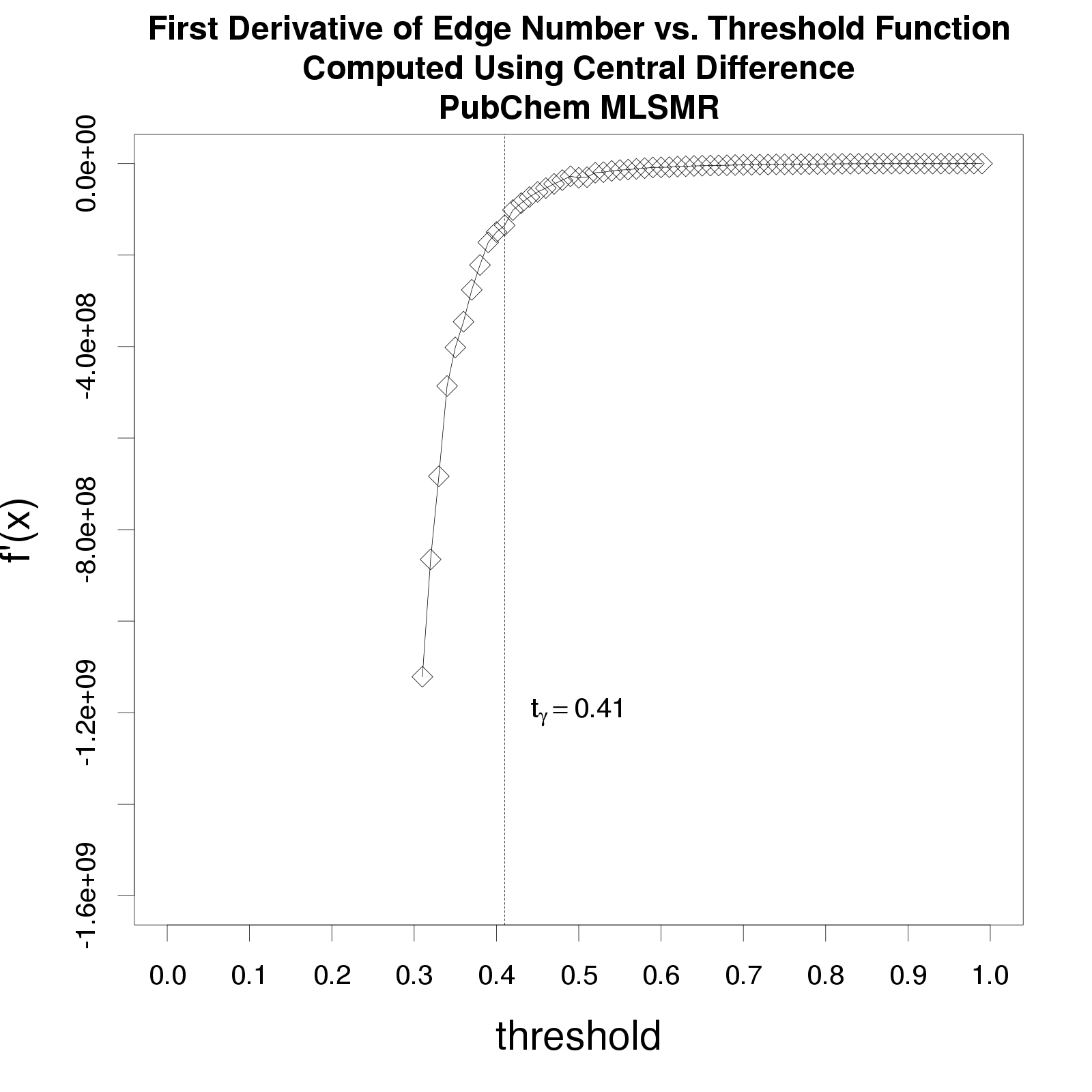


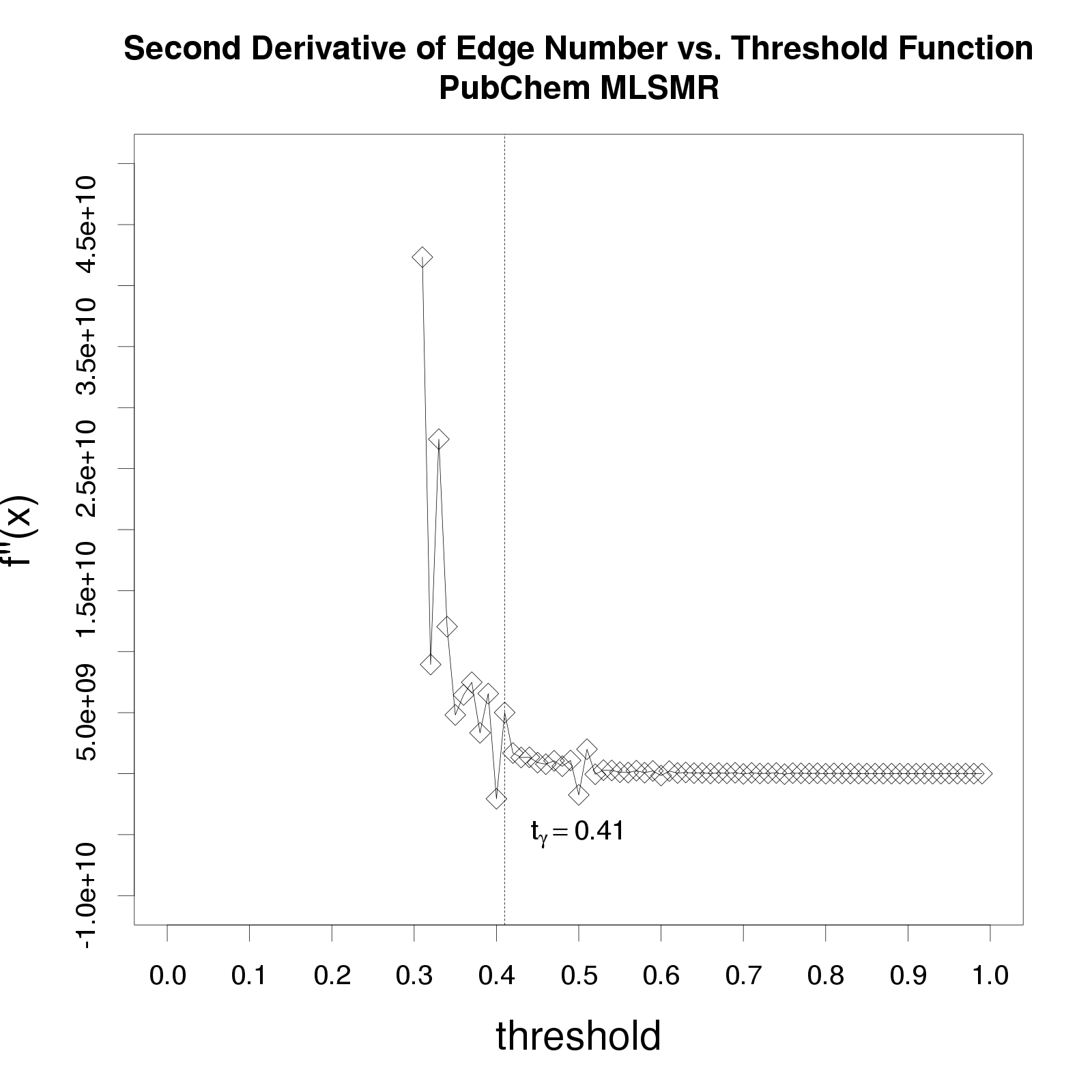

Supplement: Supplementary file 19 — 10.1186/s13321-016-0127-5 First and second order derivatives of the number of edges vs. threshold function in the case of the PubChem MLSMR dataset. The aforementioned function is denoted by f(x), and it’s first and second order derivatives by f′(x) and f″(x), respectively. The derivatives were approximated by numerical differentiation. The vertical line at threshold t γ denotes the threshold associated with the observed best clustering performance. First order derivatives: (a) using the forward difference, (b) using the backward difference, (c) using the central difference. (d) Second order derivative. [file 13321_2016_127_MOESM19_ESM.docx]
